# NARASIMHA: Novel Assay based on Targeted RNA Sequencing to Identify ChiMeric Gene Fusions in Hematological Malignancies

**DOI:** 10.1038/s41408-020-0313-6

**Published:** 2020-05-05

**Authors:** Nikhil Patkar, Prasanna Bhanshe, Sweta Rajpal, Swapnali Joshi, Shruti Chaudhary, Gaurav Chatterjee, Prashant Tembhare, Chetan Dhamne, Maya Prasad, Nirmalya Roy Moulik, Dhanalaxmi Shetty, Anant Gokarn, Avinash Bonda, Lingaraj Nayak, Sachin Punatkar, Bhausaheb Bagal, Manju Sengar, Gaurav Narula, Navin Khattry, Shripad Banavali, P. G. Subramanian, Sumeet Gujral

**Affiliations:** 1 Haematopathology Laboratory, ACTREC, Tata Memorial Centre, Navi Mumbai, India; 2 Homi Bhabha National Institute (HBNI), Mumbai, India; 3 Pediatric Haematolymphoid Disease Management Group, Tata Memorial Centre, Mumbai, India; 4 Department of Cytogenetics, ACTREC, Tata Memorial Centre, Navi Mumbai, India; 5 Adult Haematolymphoid Disease Management Group, Tata Memorial Centre, Mumbai, India

**Keywords:** Genetic testing, Cancer genomics

Dear Editor,

Chimeric gene fusions (CGF) are the hallmark of several haematological malignancies. Their exact characterization is critical for accurate diagnosis, administering targeted therapy, as well as effective post therapeutic monitoring. In that context, commonly used molecular techniques such as fluorescent in situ hybridization (FISH) are limited by low sensitivity (~5–10%), and an inherent inability to provide sequence level characterization of the chimeric gene. As compared to FISH, real-time PCR (qPCR) is more sensitive but requires a priori nucleotide level knowledge of the CGF. Furthermore, qPCR cannot be multiplexed beyond a few targets and is relatively low throughput in nature. Although transcriptome sequencing has immensely contributed towards the discovery of CGF, its applicability outside of a research setting is limited due to high sequencing costs and an impractical turn-around-time. Researchers have therefore developed focussed target enrichment strategies (or gene-panels) for detection of CGF. Typically, these panels utilize capture probes^1,2^ or multiplexed PCR approaches^3,4^ to enrich targets of interest and detect the CGF, if present. These assays too require prior knowledge of the exact sequence of both partners involved in the formation of CGF thus failing to overcome a principal hurdle of being unable to detect a CGF involving a promiscuous gene where recombination with several partners is known to occur (for e.g., *KMT2A*-rearranged leukemia)^5^. With recognition of newer entities, such as CGF driven eosinophilia^6^, *BCR–ABL1*-like ALL^6^, B-other ALL^7^ and recent precision medicine initiatives targeting *BCR–ABL1-*like ALL^8^, there is an urgent need for developing diagnostic approaches, which are within the reach of most diagnostic laboratories.

We describe NARASIMHA, a targeted RNA-sequencing assay for detection of CGF in blood cancers. NARASIMHA requires knowledge of only one of the partners involved in the formation of a CGF and can detect any potential gene fusion associated with that partner. Sample processing steps include enzymatic fragmentation of second-stranded cDNA followed by end repair, adenylation, and ligation of a novel structure called strand-specific unique molecular motif (spUMM). We designed spUMM to include an eight-base unique molecular identifier (UMI), which results in each strand of a cDNA molecule being tagged with a unique molecular fingerprint. The ligation of a spUMM creates a shotgun assembly where an incomplete semi-Y adapter is attached to each end of cDNA (Fig. 1). A unique aspect of NARASIMHA involves amplification with a primer for target enrichment from one end of cDNA while the other end is amplified with a universal primer when ligated to the spUMM. In subsequent nested PCR steps, a fully functional sequencing ready library is constructed by introduction of sample specific dual indices and instrument-specific adapters. NARASIMHA comprises of independent lymphoid and myeloid modules consisting of different primer sets for target enrichment (Supplementary Tables 1–3). Based on the clinical indication, (acute myeloid leukemia, acute lymphoblastic leukemia, chronic myeloproliferative neoplasm, MDS-MPN, or eosinophilia) we decide upon the NARASIMHA module for library preparation. Details pertaining to assay setup and performance metrics can be seen in supplementary methods accompanying this manuscript. In a linearity experiment, we could demonstrate that both modules detected CGF at a lower limit of 0.5% (Supplementary Figs. 1 and 2). In initial validation experiments, we demonstrated that NARASIMHA could detect common CGF associated with acute leukemia, such as *BCR–ABL1, RUNX1-RUNX1T1, CBFB-MYH11, ETV6-RUNX1*, and *TCF3-PBX1* (Supplemental Methods). Encouraged by this data, we prospectively tested this assay on clinical samples. We describe here a total of 107 CGF detected by NARASIMHA, which would have been difficult (or in some cases impossible) to detect using conventional techniques like FISH and conventional karyotyping. These include five novel CGF as well as other rare CGF. Clinical features, laboratory details including validation of the fusions, flow cytometric MRD, and patient outcome can be seen in Supplementary Tables 4–6 and Supplementary Fig. 3.

**Fig. 1:**
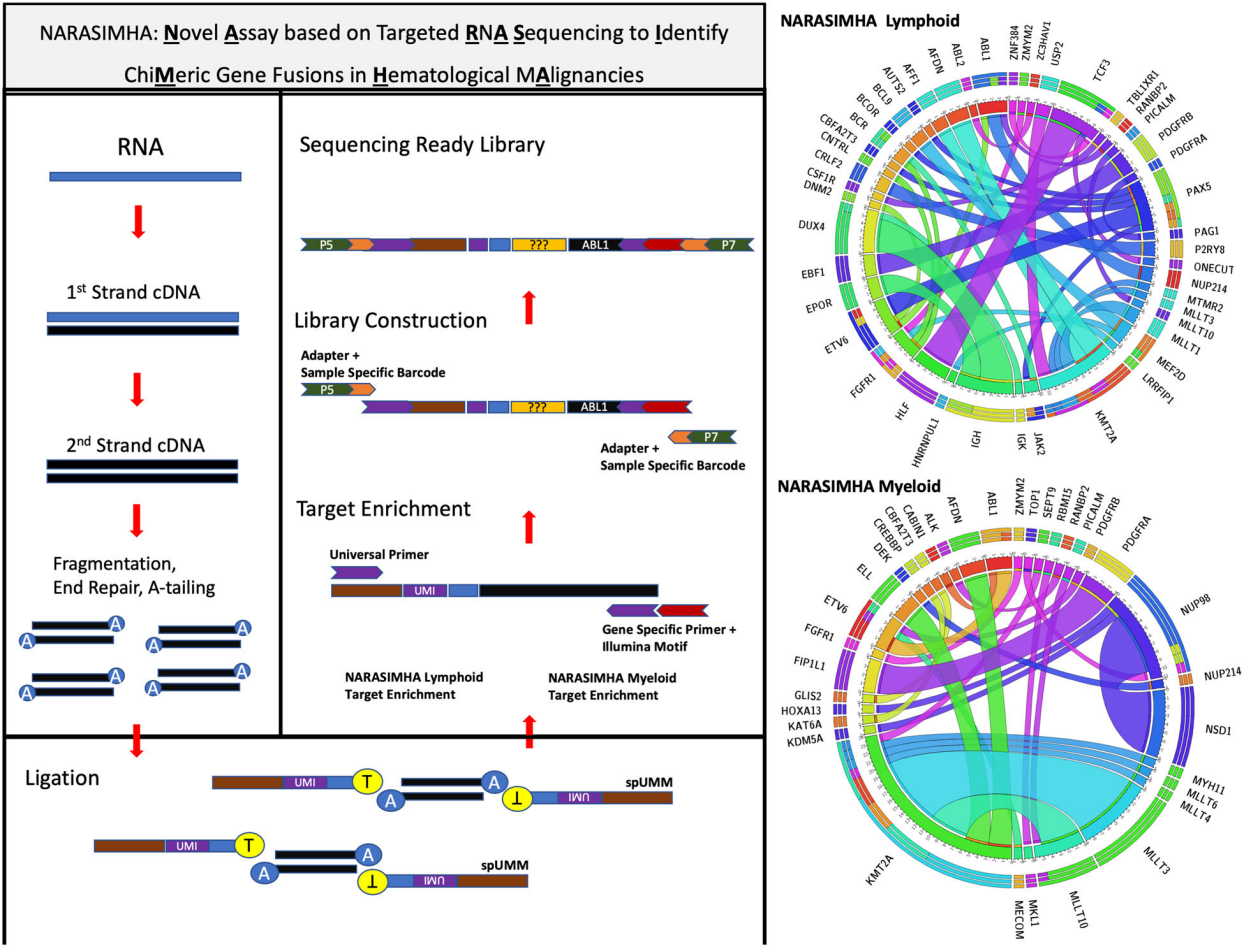
**Schematic representation of NARASIMHA. a** RNA is used as a starting template and converted to second strand DNA, enzymatically fragmented, end-repaired, and A-tailed. **b** Fragmented cDNA is ligated to strand-specific unique molecular motifs (spUMM). **c** spUMM-ligated fragments undergo target enrichment and library construction to generate a sequencing ready library. **d** Circos plot elucidates chimeric gene fusions detected using the lymphoid module of NARASIMHA. **e** Circos plot elucidates chimeric gene fusions detected using the myeloid module of NARASIMHA.

The myeloid module of NARASIMHA detected the following CGF (Supplementary Table 5):

1. AML with *KMT2A* gene rearrangement (*n* = 25): These included *KMT2A-MLLT3* (*n* = 9), *KMT2A-MLLT10* (*n* = 6), *KMT2A-AFDN* (*n* = 3), *KMT2A-ELL* (*n* = 3), and one each of *KMT2A-MLLT4, KMT2A-MLLT6, KMT2A-MYH11*, and *KMT2A-SEPT9*.
2. AML with NUP98 gene rearrangement (*n* = 10): Most commonly this included *NUP98–NSD1* (*n* = 7) followed by three others (*NUP98-HOXA13, NUP98-TOP1*, and *NUP98-KDM5A*).
3. Other rare CGF seen in AML (*n* = 5): These included entities recognized recently by the WHO classification, such as AML with *ETV6-MECOM* (*n* = 1), AML with *RBM15-MKL1* (*n* = 1), and AML with *DEK-NUP214* (*n* = 1). Other rare fusions, such as *KAT6A-CREBBP* (*n* = 1) and *CBFAT-GLIS2* (*n* = 1), were also observed.
4. Myeloproliferative neoplasms including molecular workup of unexplained eosinophilia (*n* = 9): CGF in patients with myeloproliferative neoplasms included *CABIN1-ABL1* (*n* = 1), *ETV6-ABL1* (*n* = 2), *ETV6-PDGFRB* (*n* = 1), *ZMYM2-FGFR1* (*n* = 1), and *FIP1L1-PDGFRA* (*n* = 4).
5. Miscellaneous: One patient with mixed lineage acute leukemia demonstrated *NUP98–NSD1* fusion, whereas we also observed a rare *RANBP2-ALK* CGF in a case of chronic myelomonocytic leukemia with eosinophilia (which did not have any other somatic mutation). A patient of acute leukemia of ambiguous lineage harboured *PICALM-MLLT10*.

The lymphoid module of NARASIMHA detected 51 CGF (excluding novel rearrangements described in Supplementary Table 4) as a result of which we could classify patients under these broad categories (Supplementary Table 6):

1. *BCR–ABL1-*like BCP-ALL: We detected 18 patients with *BCR–ABL1-*like *BCP-ALL*. These included cases with rearrangements of *ABL1* (*n* = 3; *NUP214, ETV6, RANBP2*), *ABL2* (*n* = 1; *ZC3HAV1*), *CRLF2* (*n* = 2; *P2RY8-CRLF2*), *EPOR* (*n* = 3; *EPOR-IGH*), *FGFR1* (*n* = 3, *BCR, CNTRL, LRRFIP1*), *JAK2* (*n* = 2; *BCR, PAX5*), *CSF1R* (*n* = 1; *TBL1XR1*), and *PDGFRB* (*n* = 3; *EBF1*).
2. B-other BCP-ALL: A total of 21 patients had CGF that could be classified as B-other BCP-ALL. Most commonly these harboured *PAX5* rearrangement [*n* = 6; *ETV6* (*n* = 3), *AUTS2, BCOR, CBFA2T3*(1 each)], *TCF3* [(*TCF3-HLF* (*n* = 5); *TCF3-ONECUT3* (*n* = 1)], *DUX4* (*n* = 5; *DUX4-IGH*), *MEF2D* [*BCL9* (*n* = 2), *HNRNPUL1* (*n* = 1)], and *ZNF384* (*n* = 1; *TCF3-ZNF384*).
3. *KMT2A*-rearranged BCP-ALL: These included *KMT2A-AFF1* (*n* = 2), *KMT2A-USP2* (*n* = 2), *KMT2A-MLLT1* (*n* = 1), and *KMT2A-MLLT3* (*n* = 1).
4. Precursor T Lineage ALL (T-ALL): We observed four patients with KMT2A rearrangements [*KMT2A-AFDN*, (*n* = 2); *KMT2A-MTMR2* (*n* = 1), *KMT2A-MLLT1*(*n* = 1)] of which three harboured an ETP-ALL immunophenotype. We also observed *ZMYM-FGFR1*, *NUP214-ABL1, IGK-DUX4, ETV6-DNM2*, and *PAG1-PDGFRA* in five patients of T-ALL (Supplementary Tables 4 and 6).
5. Miscellaneous: One cases of acute leukemia of ambiguous lineage harboured *PICALM-MLLT10* and another harboured *KMT2A-AFDN*.

Novel CGF were detected in five patients (Supplementary Table 4).

1. *MTMR2-KMT2A*: In-frame fusion was observed between exon 8 of *KMT2A* and exon 3 of the *MTMR2* gene in a 31-year-old male with ETP-ALL. This is a novel partner of the *KMT2A* recombinome.
2. *ETV6-DNM2*: Out-of-frame fusion seen between exon 2 of *ETV6* and exon 13 of *DNM2*. Novel partner of *ETV6* in a 15-year-old male with T-ALL.
3. *PAG1-PDGFRA*: Novel partner of *PDGFRA* where the 5’ UTR of PAG1 is involved in fusion with exon 10 of *PDGFRA* in a 30-year-old male with ETP-ALL.
4. *TCF3-ONECUT3*: In-frame fusion in a 10-year-old child with BCP-ALL between exon 16 of TCF3 and exon 2 of *ONECUT3*.
5. *IGK-DUX4*: *DUX4* associated translocation was seen with a novel partner (*IGK@*) in a 23-year-old male with T-ALL.

We could validate each of the above described CGF using orthogonal techniques, such as RT-PCR and/or FISH (Supplementary Tables 4–6 and Supplementary Fig. 4). A total of 35 *KMT2A* rearrangements were detected by NARASIMHA. Of these, 28 were detected as *KMT2A* rearranged by FISH (partner could not be identified in 18; partner characterized in 10). In six cases FISH missed the *KMT2A* rearrangement. In one case FISH was not performed.

Previously, Zheng et al. have previously described an anchored multiplex PCR assay that enable a user to detect CGF without prior knowledge of fusion partners^9^. As compared to Zheng’s method NARASIMHA represents a technical advance by the inclusion of an UMI. This enables us to perform absolute cDNA molecule counting and reduction of PCR bias as every molecule of cDNA is marked uniquely by a random oligonucleotide. Recently, Dillon et al. described a method for ultrasensitive MRD monitoring of CGF^10^. They performed multiplexed cDNA synthesis of a limited number of targets and incorporated a UMI using PCR after the cDNA synthesis stage. As compared to NARASIMHA, Dillon’s assay is more sensitive but is unable to detect unknown partners of CGF.

The power of NARASIMHA lies in the fact that we can detect CGF that are challenging for conventional techniques, such as *BCR–ABL1*-like ALL, B-other BCP-ALL, *KMT2A*-rearranged malignancies as well as cryptic lesions that will be missed by FISH (for e.g., *NUP98–NSD1*, *DUX4* rearrangements, *KMT2A-USP2* fusions). The clinical potential of this assay is evident from our data on prospective testing. This assay could contribute to the diagnosis of difficult cases (such as *BCR–ABL1*-negative myeloproliferative neoplasms as well as cases with unexplained eosinophilia) and enable appropriate prognostication by delineating extremely high risk acute leukemia (for e.g., *TCF3-HLF-*rearranged BCP-ALL or *NUP98–NSD1-*rearranged AML). The clinical utility of this assay in detection of *BCR–ABL1-*like ALL cannot be overstated. Nearly 93% of cases in which FCM-MRD testing was performed (13 out 14 cases; Supplementary Table 6) were high MRD positive (median 7.6%) at end of induction indicating a generally poor outcome for this disease. The singular case that was MRD-negative was treated with dasatinib in addition to conventional ALL chemotherapy. Although this is not explicitly demonstrated here, this assay has the potential to track MRD by monitoring CGF. This can be made possible by using high-throughput sequencers, such as the Illumina NextSeq 550 or beyond.

The cost of library preparation is approximately (USD) $56. The cost of sequencing is $70 for 2 million reads on a MiSeq v2- 500 cycle chemistry. The turn-around time for our test is 2 weeks. Unlike commercial assays (or for that matter even some of the previously published papers, which do not reveal their methodology)^9^, we offer an open source solution that will reduce the cost of testing and enable laboratories to develop customized solutions. Importantly, we provide an assay that could enable the implementation of precision medicine in diseases like *BCR–ABL1*-like ALL and enable laboratories to meaningfully classify diseases beyond the WHO 2017 classification. We estimate that incorporation of NARASIMHA into routine diagnostic workflows will make conventional techniques such as FISH redundant.

## Supplementary information

Supplemental Methods

## References

[1] Heyer EE (2019). Diagnosis of fusion genes using targeted RNA sequencing. Nat. Commun..

[2] Reeser JW (2017). Validation of a targeted RNA sequencing assay for kinase fusion detection in solid tumors. J. Mol. Diagn..

[3] Blidner RA (2019). Design, optimization, and multisite evaluation of a targeted next-generation sequencing assay system for chimeric RNAs from gene fusions and exon-skipping events in non-small cell lung cancer. J. Mol. Diagn..

[4] Ruminy P (2016). Multiplexed targeted sequencing of recurrent fusion genes in acute leukaemia. Leukemia.

[5] Mertens F, Johansson B, Fioretos T, Mitelman F (2015). The emerging complexity of gene fusions in cancer. Nat. Rev. Cancer.

[6] Arber DA (2016). The 2016 revision to the World Health Organization classification of myeloid neoplasms and acute leukemia. Blood.

[7] Schwab C, Harrison CJ (2018). Advances in B-cell precursor acute lymphoblastic leukemia genomics. Hemasphere.

[8] Tanasi I (2019). Efficacy of tyrosine kinase inhibitors in Ph-like acute lymphoblastic leukemia harboring ABL-class rearrangements. Blood.

[9] Zheng Z (2014). Anchored multiplex PCR for targeted next-generation sequencing. Nat. Med..

[10] Dillon LW (2019). Targeted RNA-sequencing for the quantification of measurable residual disease in acute myeloid leukemia. Haematologica.

