# Supplementary material for: NARASIMHA: Novel Assay based on Targeted RNA Sequencing to Identify ChiMeric Gene Fusions in Hematological Malignancies
Source: Blood Cancer J. 2020 May 5;10(5):50. doi: 10.1038/s41408-020-0313-6 (PMC7200652; doi:10.1038/s41408-020-0313-6)
Supplement: Supplementary file 1 — Supplemental Methods [file 41408_2020_313_MOESM1_ESM.docx]

| **NARASIMHA Lymphoid Panel (25)** | | | **NARASIMHA Myeloid Panel (28)** | | |
| --- | --- | --- | --- | --- | --- |
| **Gene** | **RefSeq ID** | **Region** | **Gene** | **RefSeq ID** | **Region** |
| *ABL1* | NM005157 | Exons 1-5 | *ABL1* | NM005157 | Exons 1-5 |
| *ABL2* | NM005158 | Exons 2-8 | *ABL2* | NM005158 | Exons 2-8 |
| *CRLF1* | NM022147 | Exon 1 | *AF4* | NM005935 | Exons 4-6,11 |
| *CRLF2* | NM022148 | Exons 1-6 | *ALK* | NM004304 | Exon 20 |
| *CSF1R* | NM005211 | Exons 9-14 | *CREBBP* | NM004380 | Exons 2-6,15 |
| *DUX4* | NM001293798 | Exon 1 | *CSF1R* | NM005211 | Exons 9-14 |
| *EBF1* | NM024007 | Exons 10-15 | *ERG* | NM182918 | Exons 7-11 |
| *EPOR* | NM000121 | Exons 7-8 | *ETV6* | NM001987 | Exons 1-6 |
| *ETV6* | NM001987 | Exons 1-6 | *FGFR1* | NM023110 | Exons 2-12,17 |
| *FGFR1* | NM023110 | Exons 2-12,17 | *GLIS2* | NM032575 | Exons 2-5 |
| *IKZF1* | NM006060 | Exons 1-3,7,8 | *IKZF3* | NM012481 | Exons 2-7 |
| *IL2RB* | NM000878 | Exon 10 | *JAK2* | NM004972 | Exons 6-13, 15-20 |
| *JAK2* | NM004972 | Exons 6-13, 15,20 | *KAT6A* | NM006766 | Exons 16,17 |
| *KMT2A* | NM005933 | Exons 2-35 | *KMT2A* | NM005933 | Exons 2-35 |
| *MEF2D* | NM005920 | Exons 5-8 | *MECOM* | NM005241 | Exons 1-4 |
| *NTRK3* | NM001007156 | Exon 15 | *MKL1* | NM020831 | Exons 4-6 |
| *NUP98* | NM016320 | Exons 12-17 | *MLLT10* | NM001195626 | Exon 15 |
| *NUP214* | NM005085 | Exons 17-19 | *MLLT3* | NM004529 | Exons 5-7,9,10 |
| *PDGFRA* | NM006206 | Exons 9-12,14 | *MLLT4/AFDN* | NM_005936 | Exon 2 |
| *PDGFRB* | NM002609 | Exons 8-9,13 | *MYH11* | NM001040113 | Exon 10,11,14-16,29,31-36 |
| *P2RY8* | NM178129 | Exon 1 | *NTRK3* | NM001007156 | Exon 14-15 |
| *PAX5* | NM016734 | Exons 1,4-8 | *NUP98* | NM016320 | Exons 8-17 |
| *RUNX1* | NM001754 | Exons 2-9 | *NUP214* | NM005085 | Exons 17-19 |
| *TCF3* | NM003200 | Exons 11-18 | *PAX5* | NM016734 | Exon 6 |
| *ZNF384* | NM001039920 | Exons 2,3,7 | *PICALM* | NM007166 | Exons 8,16-19 |
|  |  |  | *PML* | NM002675 | Exon 3 |
|  |  |  | *RUNX1* | NM001754 | Exons 2-9 |
|  |  |  | *RUNX1T1* | NM004349 | Exons 2-3 |

**NARASIMHA**: **N**ovel **A**ssaybased on Targeted **R**N**AS**equencing to **I**dentify Chi**M**eric Gene Fusions in **H**ematologicalM**A**lignancies

**Supplementary Table 1:**

**Supplementary Table 1: List of genes and their targets amplified in myeloid and lymphoid modules of NARASIMHA**

**RNA Extraction &cDNA Synthesis:**

RNA was extracted from blood or bone marrow using QIAamp RNA Blood Mini Kit (Qiagen, Hilden, Germany) on a QiaCube automated extraction system as per the manufacturer’s instructions. First strand cDNA Synthesis (500ng RNA as input and 50 μM Oligo d(T)_20_ primer) was performed using SuperScript IV Reverse Transcriptase kit as per themanufacturer’s recommendations. (Thermo Fisher Scientific, USA). The sample was then subjected to a sample integritycheck by assaying control gene(*ABL1*) copy numbers. (1) Samples with<10,000 *ABL1*copy numbers were rejected. Subsequently, second strand cDNA was synthesized using NEBNext Ultra II Non-Directional RNA Second Strand Synthesis Module (New England Biolabs, Ipswich, MA, USA).

**NARASIMHA library preparation and sequencing:**

The second strand cDNA was size selected using SPRIselectbeads (Beckman Coulter Life Sciences, Indianapolis, USA; 1.8X) and subjected to enzymatic fragmentation using NEBNext dsDNA Fragmentase for 30 minutes. Following this the template was end repaired, A tailed and ligated to spUMMs. Oligonucleotide sequences of spUMMs (upper and lower) are seen in supplementary table 2. Post spUMM ligation, the library was purified using AMPure XP beads (Beckman Coulter Life Sciences, Indianapolis, USA; 1.5X). Preliminary target enrichment was performed usingIntermed_Fusion_A (supplementary table 2) and an equimolar pool of primers for round 1 PCR (supplementary table 3) using HotStarTaq Master Mix (Qiagen, Hilden, Germany) under the following conditions; [Denaturation:95⁰C-10 minutes; Denaturation: 95⁰C-30 seconds; Annealing 60⁰C-45 seconds; Extension 72⁰C-45 seconds; 20 cycles; final extension 72⁰C-10 minutes]. PCR product was purified using AMPure XP beads (0.8X). The eluate was amplified using Intermed_Fusion_B and primer pool for round 2 PCR (supplementary table 3) under similar PCR conditions as listed above. Primers used for round 2 PCR which are listed in supplementary Table 3 were tagged with GTCTCGTGGGCTCGGAGATGTGTATAAGAGACAGto incorporate Illumina sequencing motifs. This PCR product too was purified using AMPure XP beads (0.8X) and subjected to a final PCR with R1_Forward and R2_Reverse primers listed in supplementary table 2 (similar PCR conditions as above except for an increase in annealing temperature; 65⁰C and PCR cycles; 35 cycles]. This fully functional library was purified using AMPure XP beads (0.8X) and sequenced on an Illumina MiSeq or MiniSeq(v2; 300 cycle chemistry, 7.5% PhiX spike) with approximately 2 million reads allotted per sample.

**Supplementary Table 2**

| **Name** | **Sequence** |
| --- | --- |
| spUMM_Upper | ACACTCTTTCCCTACACGACGCTCTTCCGATCTNNNNNNNNTATGGCTGCCTGT |
| spUMM_Lower | /5Phos/CAGGCAGCCATA |
| Intermed_Fusion_A | TTACTATGCCGCTGGTGGCTCTAGATGTGAGAAAGGGATGTGCTGCGAGAAGGCTAGA |
| Intermed_Fusion_B | AATGATACGGCGACCACCGAGATCTACACTCTTTCCCTACACGACGCTCTTCCGATCT |
| R1_Forward | AATGATACGGCGACCACCGAGATCTACACXXXXXXXXXX  ACACTCTTTCCCTACACGACGCTCTTCCGATCT |
| R2_Reverse | CAAGCAGAAGACGGCATACGAGATXXXXXXXXXXGTCTCGTGGGCTCGG |

**Supplementary Table 2: List of primers critical to construction of sequencing library using spUMMs, XXXXXXXXXX indicates 10 basepair sample barcode index, NNNNNNNN indicates unique molecular barcode.**

**Supplementary Table 3**

| **Name** | **Sequence for Round 1 PCR** | **Sequence for Round 2 PCR** |
| --- | --- | --- |
| ABL1_Ex1 | TCTTGGATTTGCAGCCCACC | TTGGATTTGCAGCCCACC |
| ABL1_Ex2 | CTGAGGCTCAAAGTCAGATGCTAC | GAGGCTCAAAGTCAGATGCTAC |
| ABL1_Ex3 | ACTGTTGACTGGCGTGATGTAG | TGTTGACTGGCGTGATGTAG |
| ABL1_Ex4 | AACCAACTCGGCCAGGGTGTT | CCAACTCGGCCAGGGTGTT |
| ABL1_Ex4A | TTTGTCGTGGATGGGGGACACACCATAG | GTCGTGGATGGGGGACACACCATAG |
| ABL1_Ex5 | GATCTCTTTCATGACTGCAGC | TCTCTTTCATGACTGCAGC |
| ABL2_Ex2 | CTTGTCTCCCTCAAATCCATCCTC | TGTCTCCCTCAAATCCATCCTC |
| ABL2_Ex3 | CCACCTGATAGCCTCATTTAGTGC | ACCTGATAGCCTCATTTAGTGC |
| ABL2_Ex4 | CTTCACTCCACTCACCATTCTGG | TCACTCCACTCACCATTCTGG |
| ABL2_Ex5 | GGGTGCTGGGTAGTGTAATGTT | GTGCTGGGTAGTGTAATGTT |
| ABL2_Ex5A | TTTGTCGTGGATGGGGGACACACCATAG | GTCGTGGATGGGGGACACACCATAG |
| ABL2_Ex6 | CCTTCATTACTGCAGCTTCTTTCAG | TTCATTACTGCAGCTTCTTTCAG |
| ABL2_Ex7 | CTCGGAGGTAATCCAGCAAATTC | CGGAGGTAATCCAGCAAATTC |
| ABL2_Ex8 | GCTCCAGCATGAGCAGTATAAGTG | TCCAGCATGAGCAGTATAAGTG |
| CRLF1_Ex1 | GAGGACGCAGAGCAGCAGC | GGACGCAGAGCAGCAGC |
| CSF1R_Ex9 | CCAGAGCCGTTGATGAATGTCC | AGAGCCGTTGATGAATGTCC |
| CSF1R_Ex10 | GCTCTAAGGTCTCAACAGTCAGCA | TCTAAGGTCTCAACAGTCAGCA |
| CSF1R_Ex11 | CTGGTGTGAAGAGGAACTCATCC | GGTGTGAAGAGGAACTCATCC |
| CSF1R_Ex12 | CTCCCACTTCTCGTTGTAAGGCA | CCCACTTCTCGTTGTAAGGCA |
| CSF1R_Ex13 | GATCTTCAGCTCGGACATGAGGG | TCTTCAGCTCGGACATGAGGG |
| CSF1R_Ex14 | GGTGGCTCATGATCTTCAGCTCGGACA | TGGCTCATGATCTTCAGCTCGGACA |
| DUX4_Ex1A | GCGCAACCTCTCCTAGAAAC | GCAACCTCTCCTAGAAAC |
| DUX4_Ex1B | CCCTCAGCGAGGAAGAATAC | CTCAGCGAGGAAGAATAC |
| DUX4_Ex1C | TGCCTGTGGGCCTTTACAAG | CCTGTGGGCCTTTACAAG |
| DUX4_Ex1D | CCGGAATTTCACGGACGGAC | GGAATTTCACGGACGGAC |
| DUX4_Ex1E | TGAGAAGGATCGCTTTCCAG | AGAAGGATCGCTTTCCAG |
| EBF1_Ex10 | GCAAAGGAACACCAGGCAGATT | AAAGGAACACCAGGCAGATT |
| EBF1_Ex11 | TGGTTTCCAGAGGTTACAGAAGGT | GTTTCCAGAGGTTACAGAAGGT |
| EBF1_Ex12 | CTGGTAGAAGCACTGTATGGGATG | GGTAGAAGCACTGTATGGGATG |
| EBF1_Ex13 | GGACAACTGGCCGTGAATGT | ACAACTGGCCGTGAATGT |
| EBF1_Ex14 | ACGGCTCAGCTGCCAACTC | GGCTCAGCTGCCAACTC |
| EBF1_Ex15 | ACCAGCACCAACGGGAACA | CAGCACCAACGGGAACA |
| EPOR_Ex7 | GGCCTCTTCACCACCCACAAG | CCTCTTCACCACCCACAAG |
| EPOR_Ex8A | AACCCTTATGAGAACAGCCTTATCC | CCCTTATGAGAACAGCCTTATCC |
| EPOR_Ex8B | CTGTGGCTGTACCAGAATGATG | GTGGCTGTACCAGAATGATG |
| ETV6_Ex1 | TCTGAGACTCCTGCTCAGTGTA | TGAGACTCCTGCTCAGTGTA |
| ETV6_Ex2A | CGAGGCACTGGAACATGAAGT | AGGCACTGGAACATGAAGT |
| ETV6_Ex2B | TCAGGATGGAGGAAGACTCGAT | AGGATGGAGGAAGACTCGAT |
| ETV6_Ex3A | GGAGAGCTTTGCCATTCATTTCAA | AGAGCTTTGCCATTCATTTCAA |
| ETV6_Ex3B | CTGACCAAAGAGGACTTTCGCTATC | GACCAAAGAGGACTTTCGCTATC |
| ETV6_Ex4A | CAGGGTGGAAGAATGGTGAAAAA | GGGTGGAAGAATGGTGAAAAA |
| ETV6_Ex4B | CCGGAGGTCATACTGCATCAGAAC | GGAGGTCATACTGCATCAGAAC |
| ETV6_Ex5A | TCAATGGTGGGAGGGTTATGGT | AATGGTGGGAGGGTTATGGT |
| ETV6_Ex5B | CGCCATGCCCATTGGGAGAATAG | CCATGCCCATTGGGAGAATAG |
| ETV6_Ex6 | ATCCGATGGGAGGACAAAGAAT | CCGATGGGAGGACAAAGAAT |
| FGFR1_Ex2 | CAGTTAGAGGTTGGTGACAAGGCT | GTTAGAGGTTGGTGACAAGGCT |
| FGFR1_Ex3 | ACCAGGAAGGACTCCACTTCC | CAGGAAGGACTCCACTTCC |
| FGFR1_Ex4 | CGGTTTGGTTTGGTGTTATCTGTT | GTTTGGTTTGGTGTTATCTGTT |
| FGFR1_Ex5 | CACTGGAAGGGCATTTGAACTT | CTGGAAGGGCATTTGAACTT |
| FGFR1_Ex6 | GGCACCACAGAGTCCATTATGA | CACCACAGAGTCCATTATGA |
| FGFR1_Ex7 | ATGAACTCCACGTTGCTACCCA | GAACTCCACGTTGCTACCCA |
| FGFR1_Ex8 | GTGAAGCACCTCCATCTCTTTGT | GAAGCACCTCCATCTCTTTGT |
| FGFR1_Ex10 | GATGGCCGAACCAGAAGAACC | TGGCCGAACCAGAAGAACC |
| FGFR1_Ex11 | ACGGTTGGGTTTGTCCTTGT | GGTTGGGTTTGTCCTTGT |
| FGFR1_Ex12 | TGAAGATGATCGGGAAGCATAAGA | AAGATGATCGGGAAGCATAAGA |
| FGFR1_Ex17A | CCTCACAGAGACCCACCTTCAA | TCACAGAGACCCACCTTCAA |
| FGFR1_Ex17B | TGAAGGTGGGTCTCTGTGAGG | AAGGTGGGTCTCTGTGAGG |
| IKZF1_Ex1 | CCAAAGCGCGACGCACAAATC | AAAGCGCGACGCACAAATC |
| IKZF1_Ex2 | GGGTCAAGACATGTCCCAAGTTTC | GTCAAGACATGTCCCAAGTTTC |
| IKZF1_Ex3 | GCAAAGCTCCAAGAGTGACAGAG | AAAGCTCCAAGAGTGACAGAG |
| IKZF1_Ex7 | TGCACAGGTCTTCTGCCATTTC | CACAGGTCTTCTGCCATTTC |
| IKZF1_Ex8 | GCTGTCGTAGGGCGTGTCG | TGTCGTAGGGCGTGTCG |
| IL2RB_Exon10A | CCCACCCTGGCCATCTGTCTACACCAA | CACCCTGGCCATCTGTCTACACCAA |
| IL2RB_Exon10B | TTGGTGTAGACAGATGGCCAGGGTG | TTGGTGTAGACAGATGGCCAGGG |
| JAK2_Ex6 | ACTGCCATCCCAAGACATTCTT | TGCCATCCCAAGACATTCTT |
| JAK2_Ex7 | GCATTGGCTGAATTGCTGAATAAA | ATTGGCTGAATTGCTGAATAAA |
| JAK2_Ex8 | CCTCTTGGTTTGCTTGCTTAATACTG | TCTTGGTTTGCTTGCTTAATACTG |
| JAK2_Ex9 | CTCTGTAAAGAAGTAGCACCTCCAG | CTGTAAAGAAGTAGCACCTCCAG |
| JAK2_Ex10A | TCCAGTCTGATTACCTGCTTTCTT | CAGTCTGATTACCTGCTTTCTT |
| JAK2_Ex10B | CTGTATGTACTTCGATGCAGTCCTAA | GTATGTACTTCGATGCAGTCCTAA |
| JAK2_Ex11 | TGGAAACTGTTCGCTCAGACAATATAA | GAAACTGTTCGCTCAGACAATATAA |
| JAK2_Ex12 | CAGAGGCCTACTCATATGAACCAAAT | GAGGCCTACTCATATGAACCAAAT |
| JAK2_Ex13 | GACCGTAGTCTCCTACTTCTCTTCG | CCGTAGTCTCCTACTTCTCTTCG |
| JAK2_Ex15 | GCCAACTGTTTAGCAACTTCAAGT | CAACTGTTTAGCAACTTCAAGT |
| JAK2_Ex20 | TGCTGTAGGGATTTCAGGATTTCA | CTGTAGGGATTTCAGGATTTCA |
| KMT2A_Ex2 | CGCACTCTGACTTCTTCATCTGA | CACTCTGACTTCTTCATCTGA |
| KMT2A_Ex3 | ATTTCGGTCAGAGCCACTTCTA | TTCGGTCAGAGCCACTTCTA |
| KMT2A_Ex4 | TTACCATGGGAAGAACGAGAAA | ACCATGGGAAGAACGAGAAA |
| KMT2A_Ex5 | CCCAAGTTTGGTGGTCGCAATATAA | CAAGTTTGGTGGTCGCAATATAA |
| KMT2A_Ex6 | GCCTACCTGCAGAAGCAAGCTAA | CTACCTGCAGAAGCAAGCTAA |
| KMT2A_Ex7 | CCACTCCTAGTGAGCCCAAGAA | ACTCCTAGTGAGCCCAAGAA |
| KMT2A_Ex8 | CCCGCCCAAGTATCCCTGTAAA | CGCCCAAGTATCCCTGTAAA |
| KMT2A_Ex9 | TCCACAGGATCAGAGTGGACTTTA | CACAGGATCAGAGTGGACTTTA |
| KMT2A_Ex10 | CCCAGGGTGGTTTGCTTTCTCT | CAGGGTGGTTTGCTTTCTCT |
| KMT2A_Ex11 | CACGTTTGTGGAAGGCAACATC | CGTTTGTGGAAGGCAACATC |
| KMT2A_Ex12 | ACCCCACCAAACCCACAAAGAA | CCCACCAAACCCACAAAGAA |
| KMT2A_Ex13 | ATGCACAGTGGTCTCATGATTTCT | GCACAGTGGTCTCATGATTTCT |
| KMT2A_Ex14 | ATCGCTGGGTCCATTCCAAAT | CGCTGGGTCCATTCCAAAT |
| KMT2A_Ex15 | ATTCTCGGACTACCAGCCATTT | TCTCGGACTACCAGCCATTT |
| KMT2A_Ex16 | AGCGGAAGTGTTTGAAGATGATGAC | CGGAAGTGTTTGAAGATGATGAC |
| KMT2A_Ex17 | TGCCAACGACATCGGGATTTGAT | CCAACGACATCGGGATTTGAT |
| KMT2A_Ex18 | AAGGAATCAGCTTGAGAAGGAAGT | GGAATCAGCTTGAGAAGGAAGT |
| KMT2A_Ex19 | TAGAAGGAGTCAAGAGGAAGATGG | GAAGGAGTCAAGAGGAAGATGG |
| KMT2A_Ex20 | GATGGAGGACAGCCAGAAATTAAA | TGGAGGACAGCCAGAAATTAAA |
| KMT2A_Ex21 | GTTTTGGGAGCCAAATAAAGTATCAAG | TTTGGGAGCCAAATAAAGTATCAAG |
| KMT2A_Ex22 | CCAACTCCTCTGCATCCTCCTA | AACTCCTCTGCATCCTCCTA |
| KMT2A_Ex23 | TAGACAGTGTGCGTTATGTTTGAC | GACAGTGTGCGTTATGTTTGAC |
| KMT2A_Ex24 | CTCTCCGACTGTGAAGATAAGCTC | CTCCGACTGTGAAGATAAGCTC |
| KMT2A_Ex25 | GAAAACAGGACCATTGCCCATAG | AAACAGGACCATTGCCCATAG |
| KMT2A_Ex26 | CCCAGGATTCGAACACCCAGTTAT | CAGGATTCGAACACCCAGTTAT |
| KMT2A_Ex27 | AAGCACACATTCCAGACCAAGA | GCACACATTCCAGACCAAGA |
| KMT2A_Ex28 | CAGTCCTCCCAGAAGGAGTGT | GTCCTCCCAGAAGGAGTGT |
| KMT2A_Ex29 | AAAATCCAGCAAATGAACAAGAAAGTG | AATCCAGCAAATGAACAAGAAAGTG |
| KMT2A_Ex30 | ATTTCCAGTGATGATGGCTTTCAG | TTCCAGTGATGATGGCTTTCAG |
| KMT2A_Ex31 | TCGATCAAATGCCCGCCTAAAG | GATCAAATGCCCGCCTAAAG |
| KMT2A_Ex32 | AGCCAGAGGAGGCCAATGAAC | CCAGAGGAGGCCAATGAAC |
| KMT2A_Ex33 | CCCCAATGATGAAGAAGAGGAGGAG | CCAATGATGAAGAAGAGGAGGAG |
| KMT2A_Ex34 | AAAAGACTTCTAAGGAGGCAGTTG | AAGACTTCTAAGGAGGCAGTTG |
| KMT2A_Ex35 | TGCAGGTGAGATGGTGATTGAGTATG | CAGGTGAGATGGTGATTGAGTATG |
| MEF2D_Ex5 | GCACTACAGAGGAACAGTGTG | ACTACAGAGGAACAGTGTGTC |
| MEF2D_Ex6 | GTGACCTGAACAGTGCTAACG | GACCTGAACAGTGCTAACGGA |
| MEF2D_Ex7 | CATCACTTCCCAGGCAGGAA | TCACTTCCCAGGCAGGAAAG |
| MEF2D_Ex8 | TCACTTGACTGAGGACCATTTAGA | CATCACTTGACTGAGGACCATT |
| NTRK3_Ex15 | GAATGTCCGGGAAGGCTTATTG | ATGTCCGGGAAGGCTTATTG |
| NUP98_Ex12 | CTTAGGTTGGTTGTTCCCAAACAA | TAGGTTGGTTGTTCCCAAACAA |
| NUP98_Ex13 | CTTTGACAGATCCAAATGCTTCTGC | TTGACAGATCCAAATGCTTCTGC |
| NUP98_Ex13A | CTATTGATGTGCTGCTGGAGAACA | ATTGATGTGCTGCTGGAGAACA |
| NUP98_Ex14 | TCTCTTTGATGGGCTGGATGAC | TCTTTGATGGGCTGGATGAC |
| NUP98_Ex15 | AAAATCTAGCTTCACCATCTGAATATC | AATCTAGCTTCACCATCTGAATATC |
| NUP98_Ex16 | TTCAGGATGACCGAGAAGAAATAGAA | CAGGATGACCGAGAAGAAATAGAA |
| NUP98_Ex17 | AAGGAGAGTGCATTGTCTCTGAT | GGAGAGTGCATTGTCTCTGAT |
| NUP214_Ex17 | ACCAGCAAAGCCCTCAAGTAAAG | CAGCAAAGCCCTCAAGTAAAG |
| NUP214_Ex18 | ATCATTCACATCTTGGACAGCAAAT | CATTCACATCTTGGACAGCAAAT |
| NUP214_Ex19 | CGATTGTTGGCTAGGGTGTTAAA | ATTGTTGGCTAGGGTGTTAAA |
| PDGFRA_Ex9 | GATCATCGACCAAGTCCAGAATGG | TCATCGACCAAGTCCAGAATGG |
| PDGFRA_Ex10 | GGATCTCCGTGATGATGTTTGAGA | ATCTCCGTGATGATGTTTGAGA |
| PDGFRA_Ex11 | GACAACCAGGACAATAAGTGAGATG | CAACCAGGACAATAAGTGAGATG |
| PDGFRA_Ex12 | CAAGCACTAGTCCATCTCTTGGAA | AGCACTAGTCCATCTCTTGGAA |
| PDGFRA_Ex14 | AGTTCAGACATGAGAGCTTGTTTT | TTCAGACATGAGAGCTTGTTTT |
| PDGFRB_Ex8 | GCCTCTGCCACCTTCACGC | CTCTGCCACCTTCACGC |
| PDGFRB_Ex9 | TCAGGGTGGCTCTCACTTAGC | AGGGTGGCTCTCACTTAGC |
| PDGFRB_Ex13 | ACTTTCATCGTGGCCTGAGAATGGCTC | TTTCATCGTGGCCTGAGAATGGCTC |
| P2RY8_Ex1 | CCTCTGAGCTCTCACCTGCTACTTCT | CCTCTGAGCTCTCACCTGCTACT |
| PAX5_Ex1 | AAATTATCCGACTCCTCGGACCAG | ATTATCCGACTCCTCGGACCAG |
| PAX5_Ex4 | CCAGTCCCAGCTTCCAGTCA | AGTCCCAGCTTCCAGTCA |
| PAX5_Ex5 | CGACACCAACAAGCGCAAGA | ACACCAACAAGCGCAAGA |
| PAX5_Ex6 | CAGCACTACTCAGACATCTTCACC | GCACTACTCAGACATCTTCACC |
| PAX5_Ex7A | TGACATCGGGAGCAGTGTG | ACATCGGGAGCAGTGTG |
| PAX5_Ex7B | AGATTGGCCTTCATGTCGTCCA | ATTGGCCTTCATGTCGTCCA |
| PAX5_Ex8A | CTGGACAGGGCAGCTACTCA | GGACAGGGCAGCTACTCA |
| PAX5_Ex8B | AGTAGCTGCCCTGTCCAGC | TAGCTGCCCTGTCCAGC |
| RUNX1_Ex2 | ATTTGAGTCATTTCCTTCGTACCC | TTGAGTCATTTCCTTCGTACCC |
| RUNX1_Ex3 | ATACTTGGAATGAATCCTTCTAGAGAC | ACTTGGAATGAATCCTTCTAGAGAC |
| RUNX1_Ex4 | TGCAACAAGACCCTGCCCATC | CAACAAGACCCTGCCCATC |
| RUNX1_Ex5 | TCAGGTTTGTCGGTCGAAGTG | AGGTTTGTCGGTCGAAGTG |
| RUNX1_Ex6 | CACCTACCACAGAGCCATCAAA | CCTACCACAGAGCCATCAAA |
| RUNX1_Ex6 | GTGAAGACAGTGATGGTCAGAGTG | GAAGACAGTGATGGTCAGAGTG |
| RUNX1_Ex7 | CTGAACCACTCCACTGCCTTTAAC | GAACCACTCCACTGCCTTTAAC |
| RUNX1_Ex7 | GGCTTGGTCTGATCATCTAGTTTCT | CTTGGTCTGATCATCTAGTTTCT |
| RUNX1_Ex8 | GACAACCCTCTCTGCAGAACTTT | CAACCCTCTCTGCAGAACTTT |
| RUNX1_Ex8 | GGCAATGGATCCCAGGTATTGG | CAATGGATCCCAGGTATTGG |
| RUNX1_Ex9 | GGTCGCTGAACGCTGTCAG | TCGCTGAACGCTGTCAG |
| TCF3_Ex11 | CCATCTGCATCCTCCTTCTCCTC | ATCTGCATCCTCCTTCTCCTC |
| TCF3_Ex12 | GGGGATGCCCTCGGCAAAG | GGATGCCCTCGGCAAAG |
| TCF3_Ex13 | ACTTCTCGTCCAGCCCTTCTA | TTCTCGTCCAGCCCTTCTA |
| TCF3_Ex14 | GCCTTATCGCCCAGCTACGAC | CTTATCGCCCAGCTACGAC |
| TCF3_Ex15 | CGCTGGCCTCAGGTTTCACC | CTGGCCTCAGGTTTCACC |
| TCF3_Ex16 | CGGCCTCCCGACTCCTACA | GCCTCCCGACTCCTACA |
| TCF3_Ex17 | CCACTCGGAGGAGGAGAAGAAG | ACTCGGAGGAGGAGAAGAAG |
| TCF3_Ex18 | GCTGTCTCGGTCATCCTGAACT | TGTCTCGGTCATCCTGAACT |
| ZNF384_Ex2 | TGCCGTCTTATTCCTTCTCCT | CCTGCCGTCTTATTCCTTCTC |
| ZNF384_Ex3 | CCAGAAGTACGGGTTAGAAT | GGCCAGAAGTACGGGTTAGA |
| ZNF384_Ex7 | GACTTGGAGTAGAATGTCAG | CCGACTTGGAGTAGAATGTC |
| AF4_Ex4 | ACAATGGACTTCATTGGAGTAGGTC | AATGGACTTCATTGGAGTAGGTC |
| AF4_Ex5 | AGGCGTATGTATTGCTGTCAAAGG | GCGTATGTATTGCTGTCAAAGG |
| AF4_Ex6 | TTGGGTTACAGAACTGACATGCT | GGGTTACAGAACTGACATGCT |
| AF4_Ex11 | TTGTCCAGCTGCCATTTGTTTG | GTCCAGCTGCCATTTGTTTG |
| ALK_Ex20 | GGAGCTTGCTCAGCTTGTACTC | AGCTTGCTCAGCTTGTACTC |
| CREBBP_Ex2 | GAAGCTCCGACAGTTGTTTATGTT | AGCTCCGACAGTTGTTTATGTT |
| CREBBP_Ex3 | AATGGACTTGTGTTCCCAGTTATTC | TGGACTTGTGTTCCCAGTTATTC |
| CREBBP_Ex4 | GCTCTCGTCTCTGACACTTATGAG | TCTCGTCTCTGACACTTATGAG |
| CREBBP_Ex5 | GTGTGCAGTTCTTCCAATGAGAG | GTGCAGTTCTTCCAATGAGAG |
| CREBBP_Ex6 | GGTCTATGGGATTTGGGTTACTTAAA | TCTATGGGATTTGGGTTACTTAAA |
| CREBBP_Ex15 | GGGGTAGGGACTCTGTTATCAATGC | GGTAGGGACTCTGTTATCAATGC |
| ERG_Ex7 | CATCTGAAGTCAAATGTGGAAGAGG | TCTGAAGTCAAATGTGGAAGAGG |
| ERG_Ex8 | CCGGTCCAGGCTGATCTCC | GGTCCAGGCTGATCTCC |
| ERG_Ex9 | CACTGTGGAAGGAGATGGTTGAG | CTGTGGAAGGAGATGGTTGAG |
| ERG_Ex10 | GATTTGCAAGGCGGCTACTT | TTTGCAAGGCGGCTACTT |
| ERG_Ex11 | AGGAACTGCCAAAGCTGGATCT | GAACTGCCAAAGCTGGATCT |
| ETV6_Ex1 | TCTGAGACTCCTGCTCAGTGTA | TGAGACTCCTGCTCAGTGTA |
| ETV6_Ex2A | TCAGGATGGAGGAAGACTCGAT | AGGATGGAGGAAGACTCGAT |
| ETV6_Ex2B | CGAGGCACTGGAACATGAAGT | AGGCACTGGAACATGAAGT |
| ETV6_Ex3A | CTGACCAAAGAGGACTTTCGCTATC | GACCAAAGAGGACTTTCGCTATC |
| ETV6_Ex3B | GGAGAGCTTTGCCATTCATTTCAA | AGAGCTTTGCCATTCATTTCAA |
| ETV6_Ex4A | CCGGAGGTCATACTGCATCAGAAC | GGAGGTCATACTGCATCAGAAC |
| ETV6_Ex4B | CAGGGTGGAAGAATGGTGAAAAA | GGGTGGAAGAATGGTGAAAAA |
| ETV6_Ex5A | CGCCATGCCCATTGGGAGAATAG | CCATGCCCATTGGGAGAATAG |
| ETV6_Ex5B | TCAATGGTGGGAGGGTTATGGT | AATGGTGGGAGGGTTATGGT |
| ETV6_Ex6A | ATCCGATGGGAGGACAAAGAAT | CCGATGGGAGGACAAAGAAT |
| ETV6_Ex6B | AGACGTAATCCCAAAGCAGTCTAC | CGTAATCCCAAAGCAGTCTACA |
| GLIS2_Ex2 | GGGAACTTGGAGTTCAGCAGGAAG | GAACTTGGAGTTCAGCAGGAAG |
| GLIS2_Ex3 | TTGGTGATACTCAGCTTCAGGTC | GGTGATACTCAGCTTCAGGTC |
| GLIS2_Ex4 | GGGAACTTGGAGTTCAGCAGGA | GAACTTGGAGTTCAGCAGGA |
| GLIS2_Ex5 | GGGCAGGAAGAACTGGAAGGAG | GCAGGAAGAACTGGAAGGAG |
| IKZF3_Ex2 | GGCACAGACTGCTCCTGAGTG | CACAGACTGCTCCTGAGTG |
| IKZF3_Ex3 | GGGCCTTCTCCACTGTCCACATTT | GCCTTCTCCACTGTCCACATTT |
| IKZF3_Ex4 | CATTTCCCATGGGTTCTGACTTTA | TTTCCCATGGGTTCTGACTTTA |
| IKZF3_Ex5 | GCGGAGGAGGTTACCTTTCTGAGTA | GGAGGAGGTTACCTTTCTGAGTA |
| IKZF3_Ex6 | CTCAAGGGAACTTCTCTGCTTGTA | CAAGGGAACTTCTCTGCTTGTA |
| IKZF3_Ex7 | GAGAGCTCTTTCACTTCCCATCTC | GAGCTCTTTCACTTCCCATCTC |
| KAT6A_Ex16 | AGAAGAAGATGAAGAGTCAGATGATG | AAGAAGATGAAGAGTCAGATGATG |
| KAT6A_Ex17 | AAGAGATACTGGCTTTAAGATAGGAGTG | GAGATACTGGCTTTAAGATAGGAGTG |
| MECOM_Ex1 | TGCCCTGCCTTTGGATCTCAT | CCCTGCCTTTGGATCTCAT |
| MECOM_Ex2 | AAGGTATTTCAGGGTAGTTGCCATA | GGTATTTCAGGGTAGTTGCCATA |
| MECOM_Ex3 | TTGACTGGCATCTATGCAGAACT | GACTGGCATCTATGCAGAACT |
| MECOM_Ex4 | GGCGCAATGTCTGCAACTACTCTA | CGCAATGTCTGCAACTACTCTA |
| MKL1_Ex4 | CCCTTGGCTCACCAGTTCTTCC | CTTGGCTCACCAGTTCTTCC |
| MKL1_Ex5 | CCCGCTCCAAGCTCCTTCTCTG | CGCTCCAAGCTCCTTCTCTG |
| MKL1_Ex6 | GAACGAATCTTCCGTTTGAGATAGT | ACGAATCTTCCGTTTGAGATAGT |
| MLLT10_Ex15 | CCCAGAAGACTGCTGAGGTAAGTG | CAGAAGACTGCTGAGGTAAGTG |
| MLLT3_Ex5 | TGCTGCTGCTGCTGGTATGAA | CTGCTGCTGCTGGTATGAA |
| MLLT3_Ex6 | TGGCAGGACTGGGTTGTTCAG | GCAGGACTGGGTTGTTCAG |
| MLLT3_Ex7 | CTCCACTTCATCTGATTCCTCCTC | CCACTTCATCTGATTCCTCCTC |
| MLLT3_Ex9 | ATGCCTTGTCACATTCACCATTCT | GCCTTGTCACATTCACCATTCT |
| MLLT3_Ex10 | TTCTGTGAAGCTCTACCAGTTCATC | CTGTGAAGCTCTACCAGTTCATC |
| MLLT4_Ex2 | TCACTCCATGGAACTCCAAATC | CATCACTCCATGGAACTCCAAATC |
| MYH11_Ex7 | ACCCGTGACGTCGAAGTTGAT | CCGTGACGTCGAAGTTGAT |
| MYH11_Ex8 | TGGCGAATTGCCCGTGATTT | GCGAATTGCCCGTGATTT |
| MYH11_Ex9 | TTGGAGAGGAAGGTGTAGTTGTTG | GGAGAGGAAGGTGTAGTTGTTG |
| MYH11_Ex10 | GCAGGACCGATGATACCACCTT | AGGACCGATGATACCACCTT |
| MYH11_Ex11 | TCCCATGAGGTGGCAAACTTTC | CCATGAGGTGGCAAACTTTC |
| MYH11_Ex14 | AACCAGCATTCCTCGTCCAG | CCAGCATTCCTCGTCCAG |
| MYH11_Ex15 | GTCATTCAGCGGGTCCATATTCTT | CATTCAGCGGGTCCATATTCTT |
| MYH11_Ex16 | AGCGAGCTCTCCGTCATCTTG | CGAGCTCTCCGTCATCTTG |
| MYH11_Ex29 | CCGGCCAGGTCTGCGTTCTC | GGCCAGGTCTGCGTTCTC |
| MYH11_Ex31 | GCTGGCGCAGCTTCGTAGACAC | TGGCGCAGCTTCGTAGACAC |
| MYH11_Ex32 | CCTCTTCCAGAGCTTCCACGGT | TCTTCCAGAGCTTCCACGGT |
| MYH11_Ex32 | ACGAGTTGCCGCTGGTTGTC | GAGTTGCCGCTGGTTGTC |
| MYH11_Ex34 | GAGCGCCTGCATGTTGACTT | GCGCCTGCATGTTGACTT |
| MYH11_Ex35 | TGAGCCTGCAGTTTGCGTAGCTG | AGCCTGCAGTTTGCGTAGCTG |
| MYH11_Ex36 | TCTCTGGAGGCACGGGCATC | TCTGGAGGCACGGGCATC |
| PICALM_Ex8 | ATATCACCTCTGTCAATTCCAACT | ATCACCTCTGTCAATTCCAACT |
| PICALM_Ex16 | TGGCATCGGAAATGGAACCACTA | GCATCGGAAATGGAACCACTA |
| PICALM_Ex17 | GGGGATCTAACTGGCAACCAAAGG | GGATCTAACTGGCAACCAAAGG |
| PICALM_Ex18 | TACACCAACAGGCATGATAGGATA | CACCAACAGGCATGATAGGATA |
| PICALM_Ex19 | GCCTGTCATGAGACCTCCAAACC | CTGTCATGAGACCTCCAAACC |
| PML_Ex3 | AGGACCTCAGCTCTTGCATCAC | GACCTCAGCTCTTGCATCAC |
| RUNX1_Ex2 | ATTTGAGTCATTTCCTTCGTACCC | TTGAGTCATTTCCTTCGTACCC |
| RUNX1_Ex3 | ATACTTGGAATGAATCCTTCTAGAGAC | ACTTGGAATGAATCCTTCTAGAGAC |
| RUNX1_Ex4 | TGCAACAAGACCCTGCCCATC | CAACAAGACCCTGCCCATC |
| RUNX1_Ex5A | TCAGGTTTGTCGGTCGAAGTG | AGGTTTGTCGGTCGAAGTG |
| RUNX1_Ex5B | AAATAAACAGAGGGTGCGTGTG | ATAAACAGAGGGTGCGTGTG |
| RUNX1_Ex6A | CACCTACCACAGAGCCATCAAA | CCTACCACAGAGCCATCAAA |
| RUNX1_Ex6B | GTGAAGACAGTGATGGTCAGAGTG | GAAGACAGTGATGGTCAGAGTG |
| RUNX1_Ex7A | CTGAACCACTCCACTGCCTTTAAC | GAACCACTCCACTGCCTTTAAC |
| RUNX1_Ex7B | GGCTTGGTCTGATCATCTAGTTTCT | CTTGGTCTGATCATCTAGTTTCT |
| RUNX1_Ex8A | GACAACCCTCTCTGCAGAACTTT | CAACCCTCTCTGCAGAACTTT |
| RUNX1_Ex8B | GGCAATGGATCCCAGGTATTGG | CAATGGATCCCAGGTATTGG |
| RUNX1_Ex9 | GGTCGCTGAACGCTGTCAG | TCGCTGAACGCTGTCAG |
| RUNX1T1_Ex 2 | AATGAACTGGTTCTTGGAGCTCCT | TGAACTGGTTCTTGGAGCTCCT |
| RUNX1T1_Ex 3 | GAAGAGGAAGGCCCATTGCTGAA | AGAGGAAGGCCCATTGCTGAA |

**Supplementary Table 3: List of primers for target enrichment.**

**Data Analysis:**

Samples were demultiplexed using instrument onboard software. Consensus reads were generated from the fastq files using MiNNN v1.0. To achieve this, we used -extract, -correct, -sort, -consensus and -mif2fastq functions of MiNNN. Once consensus fastq files were generated they were processed using Fusion catcher v1.23. Annotation of domains and analysis of oncogenic potential of the chimeric gene fusion was performed using Oncofuse(2). Coverage was calculated using BedTools v2.4. Visualization of the fusion was done using FusionInspector (Supplementary Figure 3) Criteria for calling a fusion were as follows:

1. Presence of at-least 5spanning reads
2. Median Coverage of at-least 20x
3. At least one of the fusion partners is a part of the enrichment panel
4. Most prevalent fusion call for that sample
5. Fusion is in-frame and at a canonical breakpoint
6. If criteria 5 is not met, then the fusion must be independently validated by an orthogonal assay

**Limit of Detection Experiment:**

We serially diluted a known *BCR-ABL1* positive sample (10%, 5%, 1%, 0.5% for the lymphoid panel) and an AML sample known to harbour *KMT2A-MLLT3* (5%, 2.5%, 1.25%, 0.63%, 0.33%) in normal cDNA. For both modules we would successfully detect the chimeric gene fusion at a limit of 0.5% & 0.63% respectively as seen in supplementary figures 1 and 2 below. Fusion percentages were calculated as (number of reads containing the fusion/number of reads mapping to that exon) X 100. All experiments were performed in duplicates from cDNA synthesis stage.

**Controls:**

Every run contained a known CGF positive case (most commonly *BCR-ABL1*) as well as a negative (normal) cDNA control.

**Supplementary Figure 1: Figure demonstrates results of a linearity experiment (lymphoid module) where a known *BCR-ABL1* positive sample was diluted in normal cDNA.**

**Supplementary Figure 2: Figure demonstrates results of a linearity experiment (myeloid module)where a known *KMT2A-MLLT3* positive sample was diluted in normal cDNA.**

**Initial Validation of Common CGF in Leukemia:**

A total of 37 samples were included in the preliminary validation step. These were patients in whom CGF were detected using FISH and conventional karyotyping and/or reverse transcription-based PCR as a part of standard genetic workup of hematolymphoid malignancies. Clinical and other laboratory features of these cases are excluded here. For the myeloid panel these included 18 cases of AML and one case of CML in blast crisis. CGF for these cases included *BCR-ABL1* (n=2), *RUNX1-RUNX1T1* (n=12), *CBFB-MYH11* (n=4). For the lymphoid panel these included 19 cases of ALL. CGF for these cases included *BCR-ABL1* (n=11), *TCF3-PBX1* (n=5), *ETV6-RUNX1* (n=3).

**Clinical Samples for Prospective Validation:**

Clinical samples were selected from routine samples submitted to the haematopathology laboratory for workup. These samples were accrued over a one-year period. Our approach to diagnosis of acute leukemia, cytogenetic workup, MRD in acute leukemia using multicolour flow cytometry have been published previously.(3-12)Patients of adult AML were treated with conventional 3+7 chemotherapy as described previously. The interim follow-up laboratory features, treatment protocol and interim follow-up are described in tables below (supplementary tables 4,5,6).

**Supplementary Table 4**

| **No** | **Fusion** | **Gene 1** | **Gene 2** | **Chromosomal Coordinates** | **Immunophenotype** | **FISH** | **Orthogonal Validation** | **Treatment** | **MRD** | **Clinical Outcome** |
| --- | --- | --- | --- | --- | --- | --- | --- | --- | --- | --- |
| 1 | *KMT2A-MTMR2* | MTMR2, exon 3 | KMT2A, exon 8 | chr11:118353210>chr11:95598840 | Early T cell Precursor T lineage Acute Lymphoblastic Leukemia | MLL translocations: t(11;?)(q23;?) in 50% cells | RT-PCR | BFM90 | Refractory Disease | Palliation |
| 2 | *ETV6-DNM2* | ETV6, exon2 | DNM2, exon13 | chr12:11905513>chr19:10916592 | Precursor T lineage Acute Lymphoblastic Leukemia | Negative for common translocations | RT-PCR | BFM90 | Post Induction FCM-MRD 0.02% | Alive (4 months follow up) |
| 3 | *TCF3-ONECUT3* | TCF3,exon 16 | ONECUT3, exon2 | chr19:1619110>chr19:1775152 | Precursor B lineage Acute Lymphoblastic Leukemia | Negative for common translocations | RT-PCR | IcIcle SR | Post Induction FCM-MRD neg | Alive (3 months follow up) |
| 4 | *PAG1-PDGFRA* | PAG1, 5'UTR | PDGFRA, exon 10 | chr8:81942231>chr4:55139704 | Early T cell Precursor T lineage Acute Lymphoblastic Leukemia | Negative for common translocations | RT-PCR | BFM90 | Post Induction FCM-MRD 78.4% | Alive (3 months follow up) |
| 5 | *IGK-DUX4* | IGK@ | DUX4, Exon1 | chr2:90310528>Un_gl000228:112467 | Precursor T lineage Acute Lymphoblastic Leukemia | TCR-A/D translocations: t(14q11) in 94% cells, 9p21 deletion: del(9p) in 92% cells | RT-PCR | IcIcle SR | Post Induction FCM-MRD neg | Alive (4 months follow up) |

**Supplementary Table 4: Table highlights genomic, immunophenotypic features and other validation details of novel fusions discovered by NARASIMHA.**

**Supplementary Table 5**

| **Sr No** | **Age** | **Sex** | **Indication/ Hematopathology Diagnosis** | **Immunophenotype/ CBC Findings** | **Karyotyping** | **FISH** | **Module** | **RNA-Sequencing** | **Other Mutations** | **Validation** | **Treatment Details** | **MRD Results** | **Outcome** | **Time to outcome (months)** | **Final Diagnosis** | **References/ Comments** |
| --- | --- | --- | --- | --- | --- | --- | --- | --- | --- | --- | --- | --- | --- | --- | --- | --- |
| **Myeloproliferative Neoplasm / Eosinophilia Under Investigation** | | | | | | | | | | | | | | | | |
| 1 | 28 | M | Myeloproliferative Neoplasm | Hb: 81 g/L;  Platelet Count: 99 x 10^9^/L;  TLC: 99.9 x 10^9^/L;  ANC: 39.9 x 10^9^/L;  AEC: 0.59 x 10^9^/L;  AMC: 0.29 x 10^9^/L | NA | BCR-ABL1 fusion | Myeloid | CABIN1-ABL1 | NA | RTPCR | TKI Therapy, Imatinib Mesylate | Attained CHR | Alive | 10 | ABL1 driven MPN | Both regions in question here (*BCR* and *CABIN1*) are adjacent genes, spaced ~900kb apart and are detected by the chr9 probe (~1.5MB). This results in a *BCR-ABL1* positive FISH pattern, but negative RT-PCR as documented by Frederick L et al. (13) It is important to recognize this entity for its monitoring implications and obvious potential pitfalls. |
| 2 | 30 | M | Myeloproliferative Neoplasm | Hb: 101 g/L;  Platelet Count: 192 x 10^9^/L;  TLC: 59.56 x 10^9^/L;  ANC: 39.9 x 10^9^/L;  AEC: 0.59 x 10^9^/L;  AMC: 4.76 x 10^9^/L | NA | 3 copies of ABL1 | Myeloid | ETV6-ABL1 | ZRSR2 (p.R295X) | RTPCR | TKI Therapy, Imatinib Mesylate | Reduction of TLC from 209 x 10^9/L to 7.54 x 10^9/L | Alive | 1 | ETV6-ABL1 driven MPN | (14) |
| 3 | 59 | M | Myeloproliferative Neoplasm | Hb: 128 g/L;  Platelet Count: 413 x 10^9^/L; TLC: 112 x 10^9^/L;  ANC: 69.44 x 10^9^/L;  AEC: 1.12 x 10^9^/L;  AMC: 3.36 x 10^9^/L | NA | 3 copies of ABL1 | Myeloid | ETV6-ABL1 | NA | RTPCR | TKI Therapy, Imatinib Mesylate subsequently switched to dasatinib and hydroxyurea | Patient attained CHR within 3 months of starting imatinib, TLC subsequently increased, Molecular MRD- 1.18% (6 months on imatinib), 0.09% | Alive | 18 | ETV6-ABL1 driven MPN |  |
| **Sr No** | **Age** | **Sex** | **Indication/ Hematopathology Diagnosis** | **Immunophenotype/ CBC Findings** | **Karyotyping** | **FISH** | **Module** | **RNA-Sequencing** | **Other Mutations** | **Validation** | **Treatment Details** | **MRD Results** | **Outcome** | **Time to outcome (months)** | **Final Diagnosis** | **References/ Comments** |
| 4 | 57 | M | Myeloproliferative Neoplasm | NA (referral case) | NA | NA | Myeloid | ETV6-PDGFRB | NA | RTPCR | NA | NA | NA | NA | Myeloid Neoplasm with PDGFRB rearrangement | (15) |
| 5 | 43 | M | Eosinophilia under evaluation | Hb: 98 g/L;  Platelet Count: 109 x 10^9^/L; TLC: 30.4 x 10^9^/L;  ANC: 7.0 x 10^9^/L;  AEC: 19.7 x 10^9^/L; | NA | NA | Myeloid | FIP1L1-PDGFRA | NA | RTPCR | NA | NA | NA | NA | CEL with FIP1L1-PDGFRA | (15) |
| 6 | 50 | M | Eosinophilia under evaluation | Hb: 108 g/L;  Platelet Count: 109 x 10^9^/L; TLC: 20.90 x 10^9^/L;  ANC: 13.33 x 10^9^/L;  AEC: 5.2 x 10^9^/L;  AMC: 1.04 x 10^9^/L | NA | CHIC2 deletion | Myeloid | FIP1L1-PDGFRA | NA | CG | TKI Therapy, Imatinib Mesylate | Bone marrow in morphological remission (no increase in eosinophils) 3 months after Imatinib | Alive | 7 | CEL with FIP1L1-PDGFRA |  |
| 7 | 72 | M | Eosinophilia under evaluation | Hb: 105.0 g/L;  Platelet Count: 103 x 10^9^/L; TLC: 17.00 x 10^9^/L;  ANC: 4.5 x 10^9^/L;  AEC: 11.05 x 10^9^/L;  AMC: 0.34 x 10^9^/L | NA | CHIC2 deletion | Myeloid | FIP1L1-PDGFRA | NA | CG | Steroids and Hydroxyurea | NA | Alive | 11 | CEL with FIP1L1-PDGFRA |  |
| **Sr No** | **Age** | **Sex** | **Indication/ Hematopathology Diagnosis** | **Immunophenotype/ CBC Findings** | **Karyotyping** | **FISH** | **Module** | **RNA-Sequencing** | **Other Mutations** | **Validation** | **Treatment Details** | **MRD Results** | **Outcome** | **Time to outcome (months)** | **Final Diagnosis** |  |
| 8 | 48 | M | Eosinophilia under evaluation | Hb: 114 .0g/L;  Platelet Count: 160 x 10^9^/L; TLC: 17.67 x 10^9^/L;  ANC: 6.7 x 10^9^/L;  AEC: 5.65 x 10^9^/L;  AMC: 0.70 x 10^9^/L | NA | FIP1L1-CHIC2-PDGFRA, Trisomy 8 | Myeloid | FIP1L1-PDGFRA | Negative | CG | TKI Therapy, Imatinib Mesylate | NA | Alive, TLC has reduced from 17.6x10^9/L to 7x10^9/L | 4 | CEL with FIP1L1-PDGFRA |  |
| 9 | 35 | M | Chronic Myelomonocytic Leukemia (CMML-2) | Hb: 98.0 g/L;  Platelet Count: 56 x 10^9^/L; TLC: 60.84 x 10^9^/L;  ANC: 41.37 x 10^9^/L;  AEC: 0.60 x 10^9^/L;  AMC: 6.69 x 10^9^/L | 45,XY,t(2;2)(p23;q13),-7[17] | Monosomy 7 | Myeloid | RANBP2-ALK | Negative | RTPCR | 6# Decitabine | Post 4# decitabine 0.17% residual myeloid blasts | Alive | 8 | RANBP2-ALK driven CMML | Known association with monocytic leukemia and monosomy 7.(16)  Novel description in CMML here |
| 10 | 23 | M | Myeloproliferative Neoplasm | Hb: 191.0 g/L;  Platelet Count: 175 x 10^9^/L; TLC: 32.86 x 10^9^/L;  ANC: 21.03 x 10^9^/L;  AEC: 3.61 x 10^9^/L;  AMC: 1.31 x 10^9^/L;  Patient had 0.22% abnormal T lymphoid blasts and 0.31% abnormal B lymphoid blasts in bone marrow at diagnosis | 46,XY,t(8;13)(p11.2;q12)[13] /46,XY[1] | FGFR1 translocation/ rearrangement | Myeloid | ZMYM2-FGFR1 | Negative | RTPCR | Planned for Modified BFM | NA | Lost to follow up | 0.5 | 8p11 Myeloproliferative Syndrome | (15, 17) |
| **Acute Myeloid Leukemia** | | | | | | | | | | | | | | | | |
| 11 | 1 | M | Acute Myeloid Leukemia | AML with monocytic maturation | NA | NA | Myeloid | KMT2A-MLLT3 | FLT3 (p.D835Y) | RTPCR | Supportive care | NA | Deceased | 0.5 | AML with KMT2A-MLLT3 | (15) |
| **Sr No** | **Age** | **Sex** | **Indication/ Hematopathology Diagnosis** | **Immunophenotype/ CBC Findings** | **Karyotyping** | **FISH** | **Module** | **RNA-Sequencing** | **Other Mutations** | **Validation** | **Treatment Details** | **MRD Results** | **Outcome** | **Time to outcome (months)** | **Final Diagnosis** | **References/ Comments** |
| 12 | 2 | M | Mixed Phenotype Acute Leukemia | Mixed Phenotype Acute Leukemia: RAM Phenotype AML M7 with NK/T cell Differentiation | NA | Negative for common translocations | Myeloid | CBFA2T3-GLIS2 | Negative | RTPCR | MCP-841 Induction Chemotherapy, Palliation | Persistent FCM-MRD positive (5.1%, 0.5%, 2.5%) | Alive | 6 | Mixed Phenotype Acute Leukemia: AML M7 with NK/T cell Differentiation | (18) |
| 13 | 33 | F | Acute Myeloid Leukemia | Acute Myeloid Leukemia with aberrant CD7 | NA | Negative for common translocations | Myeloid | DEK-NUP214 | FLT3-ITD, FLT3 (p.N676K) | RTPCR | NA | NA | Lost to follow-up |  | AML with DEK-NUP214 | (19) |
| 14 | 35 | M | Acute Myeloid Leukemia | Acute Myeloid Leukemia | NA | Negative for common translocations | Myeloid | ETV6-MECOM | GATA2(p.G320V), NF1(p.T676fs), BCOR(p.T917fs) | RTPCR | Conventional "3+7" Chemotherapy | NA | Deceased | 0.5 | AML with ETV6-MECOM | (15) |
| 15 | 16 | M | Acute Myeloid Leukemia | AML with monocytic differentiation | NA | Negative for common translocations | Myeloid | KAT6A-CREBBP | Negative | RTPCR | Conventional "3+7" Chemotherapy | FCM-MRD Negative | Relapsed | 19 | AML with KAT6A-CREBBP | (20) |
| 16 | 29 | F | Acute Myeloid Leukemia | Acute Myeloid Leukemia | NA | MLL/MLLT4 fusion: t(6;11)(q27;q23) | Myeloid | KMT2A-AFDN | NRAS(p.G13D), ASXL1(p.G867R) | CG | Conventional "3+7" Chemotherapy | Refractory Disease | Palliation, lost to follow up | 1 | AML with KMT2A-AFDN | (21) |
| 17 | 25 | M | Acute Myeloid Leukemia | AML with monocytic differentiation | NA | MLL/MLLT4 fusion: t(6;11)(q27;q23) | Myeloid | KMT2A-AFDN | Negative | CG | Conventional "3+7" Chemotherapy Planned | NA | Lost to follow up |  | AML with KMT2A-AFDN |  |
| 18 | 15 | M | Acute Myeloid Leukemia | Relapsed AML with monocytic differentiation | NA | MLL/MLLT4 fusion: t(6;11)(q27;q23) | Myeloid | KMT2A-AFDN | Negative | CG | Conventional "3+7" Chemotherapy | NA | Relapsed AML |  | AML with KMT2A-AFDN |  |
| **Sr No** | **Age** | **Sex** | **Indication/ Hematopathology Diagnosis** | **Immunophenotype/ CBC Findings** | **Karyotyping** | **FISH** | **Module** | **RNA-Sequencing** | **Other Mutations** | **Validation** | **Treatment Details** | **MRD Results** | **Outcome** | **Time to outcome (months)** | **Final Diagnosis** | **References/ Comments** |
| 19 | 33 | M | Acute Myeloid Leukemia | Aberrant CD7 | 46,XY[20] | Negative for common translocations | Myeloid | KMT2A-ELL | NRAS(p.G12D) | RTPCR | 5# Hypomethylating Agent | Post 3 cycles MRD:0.6 | Alive | 8 | AML with KMT2A-ELL | (21) |
| 20 | 26 | F | Acute Myeloid Leukemia | Acute Myeloid Leukemia | 46,XX,t(11;19)(q23;p13.1)[10]/46,XX[5] | MLL translocation t(V;11)(?;q23) | Myeloid | KMT2A-ELL | KIT(p.D816V), NRAS(p.G12A) | RTPCR | Conventional "3+7" Chemotherapy | NA | Induction Death | 0.5 | AML with KMT2A-ELL | (21) |
| 21 | 11 | F | Acute Myeloid Leukemia | AML with monocytic differentiation, aberrant CD2 expression | NA | MLL translocation t(V;11)(?;q23) | Myeloid | KMT2A-ELL | FLT3(p.D835Y) | RTPCR | 1#OMCT, 3+7 induction, 1# HIDAC, 1# Clad AraC-->OMCT | Persistent FCM-MRD positive (0.04%, 0.17%,0.05%) | Alive | 8 | AML with KMT2A-ELL |  |
| 22 | 10 | F | Acute Myeloid Leukemia | Acute Myeloid Leukemia | NA | MLL translocation t(V;11)(?;q23) | Myeloid | KMT2A-MLLT10 | KDM6A (p.S1067L) | RTPCR | Conventional "3+7" Chemotherapy | Post Induction FCM-MRD- 67% | Deceased | 2 | AML with KMT2A-MLLT10 | (21) |
| 23 | 2 | F | Acute Myeloid Leukemia | Acute Myeloid Leukemia | NA | MLL translocation t(V;11)(?;q23) | Myeloid | KMT2A-MLLT10 | CBL(p.L405_W408del), FLT3(p.M664I) | RTPCR | NO DETAILS AVAILABLE |  | NA |  | AML with KMT2A-MLLT10 |  |
| 24 | 15 | M | Acute Myeloid Leukemia | Acute Myeloid Leukemia | NA | MLL translocation t(V;11)(?;q23) | Myeloid | KMT2A-MLLT10 | PTPN11 (p.p.F71L) | RTPCR | Conventional "3+7" Chemotherapy |  | Deceased | 1 | AML with KMT2A-MLLT10 |  |
| 25 | 21 | M | Acute Myeloid Leukemia | Acute Myeloid Leukemia | NA | MLL translocation t(V;11)(?;q23) | Myeloid | KMT2A-MLLT10 | NRAS (p.G12C) | RTPCR | NA | NA | NA |  | AML with KMT2A-MLLT10 |  |
| 26 | 11 | M | Acute Myeloid Leukemia | AML with monocytic differentiation | 46,XY[15] | MLL translocation t(V;11)(?;q23) | Myeloid | KMT2A-MLLT10 | U2AF1 (p.S34F) | CG | Conventional "3+7" Chemotherapy | Post Induction FCM-MRD- Negative, Post 3# HiDAC-8.4% | Lost to follow up |  | AML with KMT2A-MLLT10 |  |
| **Sr No** | **Age** | **Sex** | **Indication/ Hematopathology Diagnosis** | **Immunophenotype/ CBC Findings** | **Karyotyping** | **FISH** | **Module** | **RNA-Sequencing** | **Other Mutations** | **Validation** | **Treatment Details** | **MRD Results** | **Outcome** | **Time to outcome (months)** | **Final Diagnosis** | **References/ Comments** |
| 27 | 2 | M | Acute Myeloid Leukemia | Acute Myeloid Leukemia with aberrant CD56 and monocytic differentiation | NA | MLL/MLLT3 fusion | Myeloid | KMT2A-MLLT3 | NRAS(p.G13D) | CG | Conventional "3+7" Chemotherapy | Post Induction FCM-MRD- Negative | Alive | 2 | AML with KMT2A-MLLT3 | (21) |
| 28 | 8 | M | Acute Myeloid Leukemia | Acute Myeloid Leukemia | 46,XY,t(9;11)(p22;q23)[7]/46,XY[3] | MLL/MLLT3 fusion, Trisomy 8 | Myeloid | KMT2A-MLLT3 | FLT3(p.I836del) | CG | Conventional "3+7" Chemotherapy | NA | Alive | 2 | AML with KMT2A-MLLT3 |  |
| 29 | 37 | M | Acute Myeloid Leukemia | Acute Myeloid Leukemia with aberrant CD56 and monocytic differentiation | NA | NEGATIVE | Myeloid | KMT2A-MLLT3 | Negative | RTPCR | Conventional "3+7" Chemotherapy | Post Induction FCM-MRD- Negative | Lost to follow up | 3 | AML with KMT2A-MLLT3 | (21) |
| 30 | 14 | F | Acute Myeloid Leukemia | Therapy related Acute monoblastic leukemia (Post choricaricinoma) | NA | MLL translocations: t(11;?)(q23;?) | Myeloid | KMT2A-MLLT3 | Negative | CG | NA | NA | Lost to follow up | 1 | AML with KMT2A-MLLT3 |  |
| 31 | 18 | M | Acute Myeloid Leukemia | AML with monocytic differentiation (CD117 and CD13 negative) | NA | MLL translocations: t(11;?)(q23;?) | Myeloid | KMT2A-MLLT3 | FLT3-TKD | CG | Conventional "3+7" Chemotherapy | Post Induction FCM-MRD- Negative | Lost to follow up | 8 | AML with KMT2A-MLLT3 |  |
| 32 | 37 | M | Acute Myeloid Leukemia | AML with monocytic differentiation (CD117 and CD13 negative) | 46,XY[16] | MLL translocations: t(11;?)(q23;?) | Myeloid | KMT2A-MLLT3 | Negative | CG | Conventional "3+7" Chemotherapy | Post Induction FCM-MRD- 5.2% | Post 3 HIDAC- CNS relapse | 14 | AML with KMT2A-MLLT3 |  |
| 33 | 42 | F | Acute Myeloid Leukemia | Therapy related AML with monocytic differentiation | NA | MLL translocations: t(11;?)(q23;?); Monosomy 7 | Myeloid | KMT2A-MLLT3 | Negative | CG | NA |  |  |  | AML with KMT2A-MLLT3 |  |
| 34 | 20 | M | Secondary AML in a treated case of B ALL | Secondary AML with monocytic differentiation | NA | Trisomy 4 | Myeloid + Lymphoid | KMT2A-MLLT3 | NA | RTPCR | BFM-90, Palliation | Post Induction FCM-MRD- Negative | Deceased | 72 | AML with KMT2A-MLLT3 |  |
| Continued….. | | | | | | | | | | | | | | | | |
| **Sr No** | **Age** | **Sex** | **Indication/ Hematopathology Diagnosis** | **Immunophenotype/ CBC Findings** | **Karyotyping** | **FISH** | **Module** | **RNA-Sequencing** | **Other Mutations** | **Validation** | **Treatment Details** | **MRD Results** | **Outcome** | **Time to outcome (months)** | **Final Diagnosis** | **References/ Comments** |
| 35 | 40 | F | Acute Myeloid Leukemia | AML with monocytic differentiation | NA | KMT2A-MLLT4 | Myeloid | KMT2A-MLLT4 | MPL(p.R592X), SETBP1(p.H210N) | CG | Conventional "3+7" Chemotherapy | NA | Relapsed | 9 | AML with KMT2A-MLLT4 | (21) |
| 36 | 12 | M | Acute Myeloid Leukemia | AML with monocytic differentiation | 46,XY[18] | MLL translocations: t(11;?)(q23;?) | Myeloid | KMT2A-MLLT6 | Negative | RTPCR | Conventional "3+7" Chemotherapy | Persistent FCM-MRD positive (0.15%, 0.05%,0.03%) | Alive | 5 | AML with KMT2A-MLLT6 | (21) |
| 37 | 34 | M | Acute Myeloid Leukemia | Acute Myeloid Leukemia | NA | MLL translocations: t(11;?)(q23;?) | Myeloid | KMT2A-MYH11 | TET2(p.R1516X), STAG2(p.S1078fs) | RTPCR | NA | NA | Lost to follow up |  | AML with KMT2A-MYH11 | (22) |
| 38 | 32 | M | Acute Myeloid Leukemia | AML with monocytic differentiation | 46,XY[20] | Negative for PML-RARA | Myeloid | KMT2A-SEPT9 | NRAS(p.G13D),KRAS(p.G13D) | RTPCR | Conventional "3+7" Chemotherapy, planned for transplant | Post Induction FCM-MRD- Negative | Lost to follow up | 1 | AML with KMT2A-SEPT9 | (21) |
| 39 | 3 | M | Acute Myeloid Leukemia | AML with monocytic differentiation, aberrant CD7, CD2 and cytoCD79a | NA | Trisomy 21 | Myeloid | NUP98-HOXA13 | NRAS(pQ61K) | RTPCR | Post Induction FCM-MRD- Negative | Post Induction FCM-MRD- Negative | Alive | 4 | AML with NUP98-HOXA13 | (23) |
| 40 | 3 | F | Acute Myeloid Leukemia | Acute Megakaryoblastic Leukemia | 46,XX[20] | Negative for common translocations; Negative for t(1;22) | Myeloid | NUP98-KDM5A | Negative | RTPCR | Conventional "3+7" Induction Chemotherapy, #2 Clad-Ara-C,palliation | Persistent FCM-MRD positive (31.6%, 0.3%,1.6%) | Palliation, Alive | 7 | AML with NUP98-KDM5A | (24) |
| Continued….. | | | | | | | | | | | | | | | | |
| **Sr No** | **Age** | **Sex** | **Indication/ Hematopathology Diagnosis** | **Immunophenotype/ CBC Findings** | **Karyotyping** | **FISH** | **Module** | **RNA-Sequencing** | **Other Mutations** | **Validation** | **Treatment Details** | **MRD Results** | **Outcome** | **Time to outcome (months)** | **Final Diagnosis** | **References/ Comments** |
| 41 | 8 | M | Acute Myeloid Leukemia | Relapsed AML with monocytic differentiation | NA | Negative | Myeloid | NUP98-NSD1 | FLT3-ITD | RTPCR | Conventional "3+7" Chemotherapy | Initial FCM-MRD positive (0.22%, 0.004%,)Post Consolidation II FCM- MRD-Negative | Relapsed | 13 | AML with NUP98-NSD1 | (25) |
| 42 | 22 | F | Acute Myeloid Leukemia | Acute Myeloid Leukemia | 47,XX,+8[13] /46,XX[1] | Trisomy 8 | Myeloid | NUP98-NSD1 | FLT3-ITD (HIGH AR), monoallelic CEBPA | RTPCR | Conventional "3+7" Chemotherapy | Post Induction FCM-MRD-90.4% | Deceased | 1 | AML with NUP98-NSD1 |  |
| 43 | 13 | F | Acute Myeloid Leukemia | Acute Myeloid Leukemia with aberrant CD7 expression | NA | Trisomy 8 | Myeloid | NUP98-NSD1 | FLT3-ITD, WT1(p.R386fs) | RTPCR | Conventional "3+7" Chemotherapy | Post Induction FCM-MRD- 0.63% | Alive | 3 | AML with NUP98-NSD1 |  |
| 44 | 12 | M | Acute Myeloid Leukemia | AML with monocytic differentiation and aberrant CD7 expression | NA | NEGATIVE | Myeloid | NUP98-NSD1 | WT1(p.R380Gfs*69) | RTPCR | NA | NA | Lost to follow-up within 1 week of starting OMCT | 1 | AML with NUP98-NSD1 | (25) |
| 45 | 43 | F | Acute Leukemia | Mixed Phenotype Acute Leukemia (B/Myeloid) | 46,XX[19] | Negative | Myeloid | NUP98-NSD1 | WT1(p.R380fs), WT1(p.R370fs),NRAS(p.G12D) | RTPCR | Conventional "3+7" Induction Chemotherapy, Reinduction with CLAG, Allogenic HSCT | Post Induction FCM-MRD- 23.6%, Post-reinduction MRD-Negative | Alive | 9 | AML with NUP98-NSD1 |  |
| 46 | 12 | M | Acute Myeloid Leukemia | AML with monocytic differentiation and aberrant CD7 expression | NA | Negative | Myeloid | NUP98-NSD1 | FLT3-ITD, NRAS(p.G13D) | RTPCR | Conventional "3+7" Chemotherapy Planned | NA | Deceased | 1 | AML with NUP98-NSD1 |  |
| **Sr No** | **Age** | **Sex** | **Indication/ Hematopathology Diagnosis** | **Immunophenotype/ CBC Findings** | **Karyotyping** | **FISH** | **Module** | **RNA-Sequencing** | **Other Mutations** | **Validation** | **Treatment Details** | **MRD Results** | **Outcome** | **Time to outcome (months)** | **Final Diagnosis** |  |
| 47 | 26 | M | Acute Myeloid Leukemia | AML with monocytic differentiation and aberrant CD19 expression | NA | trisomy 8, 22 and 3 copies of BCR | Myeloid | NUP98-NSD1 | FLT3-ITD, RUNX1 | RTPCR | Conventional "3+7" Induction Chemotherapy, Palliation | Post Induction FCM-MRD- 68% | Deceased | 2 | AML with NUP98-NSD1 |  |
| 48 | 40 | M | Acute Myeloid Leukemia | AML with monocytic differentiation and aberrant CD7 expression | NA | NA | Myeloid | NUP98-NSD1 | FLT3-ITD | RTPCR | MCP-841 Induction Chemotherapy, Palliation | Post Induction FCM-MRD-0.6%, Post Induction FCM-MRD-47% | Alive | 2 | AML with NUP98-NSD1 |  |
| 49 | 6 | M | Acute Myeloid Leukemia | Acute Myeloid Leukemia with aberrant CD7 expression | NA | Negative | Myeloid | NUP98-TOP1 | NRAS(p.Q61H), STAG2(p.L961fs) | RTPCR | Conventional "3+7" Chemotherapy | Post Induction FCM-MRD- Negative | Patient progressed from AML to MPAL (ETP ALL/Myeloid)--- Lost to follow up | 13 | AML with NUP98-TOP1 | (26) |
| 50 | 30 | M | Acute Leukemia | Acute leukemia of ambiguous lineage. Blasts express CD33, CD117, HLADR, CD56 and CD123 | NA | Negative | Myeloid | PICALM-MLLT10 | Monoallelic CEBPA, NRAS(p.G13D), PHF6(p.R24Vfs*11) | RTPCR | BFM-90 | Post Induction FCM-MRD- 0.37% | Alive | 2 | Acute Leukemia of Ambiguous lineage with PICALM-MLLT10 | (27) |
| 51 | 1 | F | Acute Myeloid Leukemia | Acute Megakaryoblastic Leukemia | 50~52,XX,t(1;22)(p13;q13),+6,+7,+11,+17,+18,+19,+21,+22[cp13] | RBM15/MKL1 fusion | Myeloid | RBM15-MKL1 | Negative | CG | Conventional "3+7" Chemotherapy | NA | Induction Death | 0.5 | AML with RBM15-MKL1 | (24) |

**Supplementary Table 5: Table highlights genomic, immunophenotypic features and other validation details of fusions discovered by the myeloid module of NARASIMHA. RT-PCR: reverse transcription-based PCR, MRD: measurable residual disease, AML: acute myeloid leukemia, CG: Conventional Karyotyping, OMCT: oral metronomic chemotherapy, NA: Not available, Hb: Hemoglobin; TLC: Total Leucocyte Count; ANC: Absolute Neutrophil Count; AEC: Absolute Eosinophil Count; AMC: Absolute Monocyte Count.**

**Supplementary Table 6**

| **Sr No** | **Age** | **Sex** | **Indication/ Hematopathology Diagnosis** | **Immunophenotype** | **Karyotyping** | **FISH** | **Module** | **RNA-Sequencing** | **Validation** | **Treatment Details** | **MRD Results** | **Outcome** | **Time to outcome (months)** | **Final Diagnosis** | **References** |  |
| --- | --- | --- | --- | --- | --- | --- | --- | --- | --- | --- | --- | --- | --- | --- | --- | --- |
| **Chimeric Gene Fusions Detected in Precursor T Lineage ALL** | | | | | | | | | | | | | | | | |
| 1 | 11 | M | Acute Lymphoblastic Leukemia | T Cell Precursor ALL | NA | TCR-B translocations: t(7q34), Monosomy 11 | Lymphoid | NUP214-ABL1 | RT-PCR | IcIcle | NA | Deceased | 1 | T Cell Precursor ALL | (28, 29) |  |
| 2 | 33 | M | Acute Lymphoblastic Leukemia | T Cell Precursor ALL | 46,XY,t(8;13)(p11.2;q12)[15]/46,XY[1] | FGFR1 rearrangement | Lymphoid | ZMYM2-FGFR1 | RT-PCR | BFM90 | Post Induction FCM-MRD 0.05% | Alive | 6 | T Cell Precursor ALL | (15) |  |
| 3 | 29 | M | Acute Lymphoblastic Leukemia | T Cell Precursor ALL | NA | MLL/MLLT4 fusion | Lymphoid | KMT2A-AFDN | CG | NA | NA | NA | NA | T Cell Precursor ALL | (21) |  |
| 4 | 27 | M | Acute Lymphoblastic Leukemia | Early T-cell Precursor T Lymphoblastic Leukemia | NA | MLL translocations: t(11;?)(q23;?) | Lymphoid | KMT2A-AFDN | RT-PCR | BFM90 | Post Induction FCM-MRD 6.5% Post Consolidation FCM-MRD 0.09% | Alive | 8 | T Cell Precursor ALL, ETP Immunophenotype | (21) |  |
| 5 | 20 | F | Acute Lymphoblastic Leukemia | Early T-cell Precursor T Lymphoblastic Leukemia | NA | MLL/MLLT1 fusion: t(11;19)(q23;p13.3) | Lymphoid | KMT2A-MLLT1 | CG | BFM90 | Post Induction FCM-MRD 17.2% Post Consolidation FCM-MRD 7.1% | Alive | 4 | T Cell Precursor ALL, ETP Immunophenotype |  |  |
| **KMT2A Rearranged BCell Precursor-ALL** | | | | | | | | | | | | | | | | |
| 6 | 19 | M | Acute Lymphoblastic Leukemia | B Cell Precursor ALL, CD10 Negative, aberrant CD15, CD33, NG2 Positive | 47, XY, +X | MLL/MLLT2 fusion: t(4;11)(q21;q23) | Lymphoid | KMT2A-AFF1 | CG | BFM90 | Post Induction FCM-MRD Negative | Alive | 8 | KMT2A Rearranged B-ALL | (21) |  |
| 7 | 49 | M | Acute Lymphoblastic Leukemia | B Cell Precursor ALL, CD10 Negative, CD15 | NA | MLL translocation in 60% cells and 1copy of MLL allele / monosomy of 11 in 30% cells | Lymphoid | KMT2A-AFF1 | CG | BFM90, CLAG induction after relapse | Post Induction FCM-MRD 0.01% | Relapsed and deceased | 5 | KMT2A Rearranged B-ALL |  |  |
| **Sr No** | **Age** | **Sex** | **Indication/ Hematopathology Diagnosis** | **Immunophenotype** | **Karyotyping** | **FISH** | **Module** | **RNA-Sequencing** | **Validation** | **Treatment Details** | **MRD Results** | **Outcome** | **Time to outcome (months)** | **Final Diagnosis** | **References** |  |
| 8 | 32 | M | Acute Lymphoblastic Leukemia | B Cell Precursor ALL, CD10 Negative, aberrant CD15, CD33, NG2 Positive | NA | MLL translocations: t(11;?)(q23;?) | Lymphoid | KMT2A-MLLT1 | RT-PCR | BFM90 | Post Induction FCM-MRD Negative | Alive | 8 | KMT2A Rearranged B-ALL | (21) |  |
| 9 | 2 | M | Acute Lymphoblastic Leukemia | B Cell Precursor ALL, CD10 Negative, CD15, CD33 Positive | NA | MLL translocations: t(11;?)(q23;?) | Lymphoid | KMT2A-MLLT3 | CG | IcIcle | Post Induction FCM-MRD Negative | Alive | 3 | KMT2A Rearranged B-ALL | (21) |  |
| 10 | 6 | M | Acute Lymphoblastic Leukemia | B Cell Precursor ALL, CD10 Negative, aberrant CD15, CD7 expressed in small subset, NG2 Positive | NA | Loss of ETV6 | Lymphoid | KMT2A-USP2 | RT-PCR | IcIcle | Post Induction FCM-MRD 89% Post Consolidation FCM-MRD 87% | Deceased | 4 | KMT2A Rearranged B-ALL | (21) |  |
| 11 | 3 | M | Acute Lymphoblastic Leukemia | B Cell Precursor ALL, CD10 Negative, aberrant CD15, CD64, NG2 Positive | NA | Negative for common translocations | Lymphoid | KMT2A-USP2 | RT-PCR | IcIcle | Post Induction FCM-MRD 0.28% Post Consolidation FCM-MRD Negative | Alive | 8 | KMT2A Rearranged B-ALL | (21) |  |
| **B-Other Subtype of BCell Precursor-ALL** | | | | | | | | | | | | | | | | |
| 12 | 3 | M | Acute Lymphoblastic Leukemia | B Cell Precursor ALL | NA | Negative for common translocations | Lymphoid | DUX4-IGH | RT-PCR | IcIcle | Post Induction FCM-MRD-Negative | Alive | 5 | B Cell Precursor ALL with DUX4-IGH | (30) |  |
| 13 | 11 | F | Acute Lymphoblastic Leukemia | B Cell Precursor ALL, aberrant CD2 | NA | IgH deletion/rearrangement | Lymphoid | DUX4-IGH | RT-PCR | IcIcle | Post Induction FCM-MRD 0.02% | Alive | 2 | B Cell Precursor ALL with DUX4-IGH |  |  |
| 14 | 6 | M | Acute Lymphoblastic Leukemia | B Cell Precursor ALL | NA | Negative for common translocations | Lymphoid | DUX4-IGH | RT-PCR | IcIcle | Post Induction FCM-MRD Negative | Alive | 2 | B Cell Precursor ALL with DUX4-IGH |  |  |
| 15 | 3 | M | Acute Lymphoblastic Leukemia | B Cell Precursor ALL | NA | Negative for common translocations | Lymphoid | DUX4-IGH | RT-PCR | IcIcle | Post Induction FCM-MRD 0.003% | Alive | 2 | B Cell Precursor ALL with DUX4-IGH |  |  |
| 16 | 6 | F | Acute Lymphoblastic Leukemia | B Cell Precursor ALL | NA | Negative for common translocations | Lymphoid | DUX4-IGH | RT-PCR | IcIcle HR | Post Induction FCM-MRD 1.04% Post Consolidation FCM-MRD Negative | Relapsed | 13 | B Cell Precursor ALL with DUX4-IGH |  |  |
| **Sr No** | **Age** | **Sex** | **Indication/ Hematopathology Diagnosis** | **Immunophenotype** | **Karyotyping** | **FISH** | **Module** | **RNA-Sequencing** | **Validation** | **Treatment Details** | **MRD Results** | **Outcome** | **Time to outcome (months)** | **Final Diagnosis** | **References** |  |
| 17 | 16 | F | Acute Lymphoblastic Leukemia | B Cell Precursor ALL, CD10 dim | NA | Negative for common translocations | Lymphoid | MEF2D-BCL9 | RT-PCR | BFM90 | Post Induction FCM-MRD 0.03% Post Consolidation FCM-MRD Negative | Relapse | 7 | B Cell Precursor ALL with MEF2D-BCL9 | (31) |  |
| 18 | 11 | F | Acute Lymphoblastic Leukemia | B Cell Precursor ALL, CD10 dim | 46,XX,t(6;10)(p23;q26)[20] | Negative for common translocations | Lymphoid | MEF2D-BCL9 | RT-PCR | IcIcle | Post Induction FCM-MRD-Negative | Alive | 3 | B Cell Precursor ALL with MEF2D-BCL9 | (31) |  |
| 19 | 3 | M | Acute Lymphoblastic Leukemia | B Cell Precursor ALL | NA | Negative for common translocations | Lymphoid | MEF2D-HNRNPUL1 | RT-PCR | IcIcle IR | Post Induction FCM-MRD-Negative | Alive | 3 | B Cell Precursor ALL with MEF2D-HNRNPUL1 | (32) |  |
| 20 | 7 | M | Acute Lymphoblastic Leukemia | B Cell Precursor ALL | NA | Negative for common translocations | Lymphoid | PAX5-AUTS2 | RT-PCR | IcIcle IR | NA | Deceased | 1 | B Cell Precursor ALL with PAX5-AUTS2 | (33) |  |
| 21 | 13 | M | Acute Lymphoblastic Leukemia | B Cell Precursor ALL | NA | Negative for common translocations | Lymphoid | PAX5-BCOR | RT-PCR | IcIcle IR | Post Induction FCM-MRD-86% | Refractory disease | 1 | B Cell Precursor ALL with PAX5-BCOR | (31) |  |
| 22 | 17 | M | Acute Lymphoblastic Leukemia | B Cell Precursor ALL | NA | loss of BCR allele & ABL1 allele, monosomy 4 and 17 | Lymphoid | PAX5-CBFA2T3 | RT-PCR | mBFM90+R | NA | Lost to follow-up | 0.5 | B Cell Precursor ALL with PAX5- CBFA2T3 | (34) |  |
| 23 | 44 | M | Acute Lymphoblastic Leukemia | B Cell Precursor ALL | NA | Negative for common translocations | Lymphoid | PAX5-ETV6 | RT-PCR | BFM90 | NA | Lost to follow-up | 0.5 | B Cell Precursor ALL with PAX5-ETV6 | (35) |  |
| 24 | 39 | M | Acute Lymphoblastic Leukemia | B Cell Precursor ALL | NA | Negative for common translocations | Lymphoid | PAX5-ETV6 | RT-PCR | No details available | NA | Lost to follow-up | NA | B Cell Precursor ALL with PAX5-ETV6 |  |  |
| 25 | 12 | F | Acute Lymphoblastic Leukemia | B Cell Precursor ALL | NA | NA | Lymphoid | PAX5-ETV6 | RT-PCR | IcIcle HR | Post Induction FCM-MRD-Negative | Alive | 3 | B Cell Precursor ALL with PAX5-ETV6 |  |  |
| 26 | 21 | M | Acute Lymphoblastic Leukemia | B Cell Precursor ALL, CD10 Negative, Aberrant CD13, 33 expression | NA | t(?;19)(?;p13) other than t(1;19) | Lymphoid | TCF3-ZNF384 | RT-PCR | BFM90 | Persistent FCM-MRD Positive (3.5, 1.7, 1.7, 0.3, 0.3%) | Alive | 20 | B Cell Precursor ALL with TCF3-ZNF384 | (36) |  |
| **Sr No** | **Age** | **Sex** | **Indication/ Hematopathology Diagnosis** | **Immunophenotype** | **Karyotyping** | **FISH** | **Module** | **RNA-Sequencing** | **Validation** | **Treatment Details** | **MRD Results** | **Outcome** | **Time to outcome (months)** | **Final Diagnosis** | **References** |  |
| 27 | 41 | F | Acute Lymphoblastic Leukemia | B Cell Precursor ALL, aberrant CD33 | NA | Negative for common translocations | Lymphoid | TCF3-HLF | RT-PCR | BFM90 | NA | Lost to follow-up | 0.5 | B Cell Precursor ALL with TCF3-HLF | (37) |  |
| 28 | 12 | M | Acute Lymphoblastic Leukemia | B Cell Precursor ALL, aberrant CD33 | NA | loss of BCR allele, trisomy 22 | Lymphoid | TCF3-HLF | RT-PCR | IcIcle | NA | Deceased | 1 | B Cell Precursor ALL with TCF3-HLF |  |  |
| 29 | 8 | F | Acute Lymphoblastic Leukemia | B Cell Precursor ALL, aberrant CD33 | NA | Negative for common translocations | Lymphoid | TCF3-HLF | RT-PCR | IcIcle, augmented BFM90, followed by TACL-bortezomib based induction | Persistent FCM-MRD Positive (18, 3.5, 10%) | Deceased | 12 | B Cell Precursor ALL with TCF3-HLF |  |  |
| 30 | 10 | M | Acute Lymphoblastic Leukemia | B Cell Precursor ALL, aberrant CD33 | NA | 3 copies of ABL1 allele | Lymphoid | TCF3-HLF | RT-PCR | IcIcle | Post Induction FCM-MRD 1.1%, Post Consolidation FCM-MRD 0.009% | Isolated medullary relapse | 12 | B Cell Precursor ALL with TCF3-HLF |  |  |
| 31 | 7 | F | Acute Lymphoblastic Leukemia | B Cell Precursor ALL, aberrant CD33 | NA | Negative for common translocations | Lymphoid | TCF3-HLF | RT-PCR | IcIcle HR | Post Induction FCM-MRD 6.3% Post Consolidation FCM-MRD 0.22% | Relapsed | 5 | B Cell Precursor ALL with TCF3-HLF |  |  |
| ***BCR-ABL1* like BCP-ALL** | | | | | | | | | | | | | | | | |
| 32 | 46 | M | Acute Lymphoblastic Leukemia | B Cell Precursor ALL, aberrant CD33, CD25 | NA | ABL1 rearrangement | Lymphoid | ETV6-ABL1 | RT-PCR | BFM90 with Dasatinib | Post Induction FCM-MRD 0.01% Post Consolidation FCM-MRD Negative | Alive | 11 | BCR-ABL1 Like ALL | (38) |  |
| 33 | 13 | M | Acute Lymphoblastic Leukemia | B Cell Precursor ALL, aberrant CD25 | NA | Negative for common translocations | Lymphoid | NUP214-ABL1 | RT-PCR | IcIcle with Imatinib | Persistent FCM-MRD Positive (10.5, 0.19, 0.18%) | Alive | 5 | BCR-ABL1 Like ALL | (28, 29) |  |
| 34 | 12 | M | Acute Lymphoblastic Leukemia | B Cell Precursor ALL, dim CD33, CD7 expression | NA | Negative for common translocations | Lymphoid | RANBP2-ABL1 | RT-PCR | IcIcle HR with Imatinib | Post Induction FCM-MRD 2.7% Post Consolidation FCM-MRD 0.001% | Alive | 5 | BCR-ABL1 Like ALL | (39) |  |
| **Sr No** | **Age** | **Sex** | **Indication/ Hematopathology Diagnosis** | **Immunophenotype** | **Karyotyping** | **FISH** | **Module** | **RNA-Sequencing** | **Validation** | **Treatment Details** | **MRD Results** | **Outcome** | **Time to outcome (months)** | **Final Diagnosis** | **References** |  |
| 35 | 20 | M | Acute Lymphoblastic Leukemia | B Cell Precursor ALL, aberrant CD33, CD13 | NA | t(1;19) | Lymphoid | ZC3HAV1-ABL2 | RT-PCR | BFM90 with Imatinib | Post Induction FCM-MRD 1.55% Post Consolidation FCM-MRD 0.55% | Alive | 9 | BCR-ABL1 Like ALL | (40, 41) |  |
| 36 | 2 | F | Acute Lymphoblastic Leukemia | NA | NA | NA | Lymphoid | P2RY8-CRLF2 | RT-PCR | IcIcle | NA | Deceased | 0.5 | BCR-ABL1 Like ALL | (30, 31) |  |
| 37 | 23 | M | Acute Lymphoblastic Leukemia | B Cell Precursor ALL, aberrant CRLF2, CD25 | NA | CRLF2 rearrangement: (Xp22) (Yp11), IKZF1 deletion | Lymphoid | P2RY8-CRLF2 | RT-PCR | BFM90 | Post Induction FCM-MRD Negative | Alive | 1 | BCR-ABL1 Like ALL | (30, 31) |  |
| 38 | 37 | F | Acute Lymphoblastic Leukemia | B Cell Precursor ALL | NA | NA | Lymphoid | EPOR-IGH | RT-PCR | BFM90 | Post Induction FCM-MRD 54.3% | Alive | 3 | BCR-ABL1 Like ALL | (42) |  |
| 39 | 17 | F | Acute Lymphoblastic Leukemia | B Cell Precursor ALL, aberrant CD33, CD25 | NA | Negative for common translocations | Lymphoid | EPOR-IGH | RT-PCR | BFM90 | Persistent FCM-MRD Positive (12.5, 1.74, 0.34, 0.1%) | Alive | 6 | BCR-ABL1 Like ALL |  |  |
| 40 | 17 | F | Acute Lymphoblastic Leukemia | B Cell Precursor ALL | NA | NA | Lymphoid | EPOR-IGH | RT-PCR | BFM90 | NA | NA | NA | BCR-ABL1 Like ALL |  |  |
| 41 | 53 | M | Acute Lymphoblastic Leukemia | B Cell Precursor ALL | 46,XY, der(8)t(8;22)(p21;q13) | Negative for common translocations | Lymphoid | BCR-FGFR1 | RT-PCR | NA | NA | NA | NA | BCR-ABL1 Like ALL | (43) |  |
| 42 | 27 | M | Acute Lymphoblastic Leukemia | Mixed Phenotype Acute Leukemia(B/T) | 50~51,XY,t(8;9)(p21;q33),+11,+12,+19,+21,+22[cp10] | Trisomy 11, 21 & 22 | Lymphoid | CNTRL-FGFR1 | RT-PCR | BFM90 | Post Induction FCM-MRD 20.7% | Alive | 4 | BCR-ABL1 Like ALL | (44) |  |
| 43 | 32 | M | Acute Lymphoblastic Leukemia | B Cell Precursor ALL, aberrant CD33, CD25 | NA | Negative for common translocations | Lymphoid | LRRFIP1-FGFR1 | RT-PCR | BFM90 | Post Induction FCM-MRD 63% Post Consolidation FCM-MRD 18% | Alive | 7 | BCR-ABL1 Like ALL |  |  |
| **Sr No** | **Age** | **Sex** | **Indication/ Hematopathology Diagnosis** | **Immunophenotype** | **Karyotyping** | **FISH** | **Module** | **RNA-Sequencing** | **Validation** | **Treatment Details** | **MRD Results** | **Outcome** | **Time to outcome (months)** | **Final Diagnosis** | **References** |  |
| 44 | 31 | M | Acute Lymphoblastic Leukemia | B Cell Precursor ALL, aberrant CD33, CD25 | NA | Monosomy 10 | Lymphoid | BCR-JAK2 | RT-PCR | BFM90 | Persistent FCM-MRD Positive (2.1, 1.8, 16.6%) | Relapse | 11 | BCR-ABL1 Like ALL | (45) |  |
| 45 | 13 | M | Acute Lymphoblastic Leukemia | B Cell Precursor ALL, dim CD33 expression | NA | PDGFRB rearrangement | Lymphoid | EBF1-PDGFRB | RT-PCR | IcIcle HR | Post Induction FCM-MRD 13.2% | Deceased | 2 | BCR-ABL1 Like ALL | (46) |  |
| 46 | 12 | M | Acute Lymphoblastic Leukemia | B Cell Precursor ALL, aberrant CD33, CD25 | NA | Negative for common translocations | Lymphoid | EBF1-PDGFRB | RT-PCR | IcIcle HR with Imatinib | Post Induction FCM-MRD 11% Post Consolidation FCM-MRD Negative | Alive | 5 | BCR-ABL1 Like ALL |  |  |
| 47 | 18 | M | Acute Lymphoblastic Leukemia | NA | NA | PDGFR-B rearranged | Lymphoid | EBF1-PDGFRB | RT-PCR | IcIcle HR with Imatinib | Persistent FCM-MRD Positive (4.7, 1.54, 0.3, 0.001%) | Alive | 38 | BCR-ABL1 Like ALL |  |  |
| 48 | 11 | M | Acute Lymphoblastic Leukemia | B Cell Precursor ALL | NA | IGH translocation | Lymphoid | PAX5-JAK2 | RT-PCR | IcIcle HR | Post Induction FCM-0.3% | Alive | 1 | BCR-ABL1 Like ALL | (46) |  |
| 49 | 48 | F | Acute Lymphoblastic Leukemia | B Cell Precursor ALL, aberrant CD33, CD25 | NA | IGH translocation, PDGFR-B rearrangement, IKZF1 deletion | Lymphoid | TBL1XR1-CSF1R | RT-PCR | BFM | NA | Alive | 0.8 | BCR-ABL1 Like ALL | (31) |  |
| **Acute Leukemia of Ambiguous lineage** | | | | | | | | | | | | | | | | |
| 50 | 15 | M | Acute Leukemia | Acute Leukemia of Ambigugous Lineage | NA | NA | Lymphoid | PICALM-MLLT10 | RT-PCR | MCP-841 | Post Induction FCM-MRD 1.1% Post Consolidation FCM-MRD 0.05% | Alive | 4 | Acute Leukemia of Ambiguous lineage with PICALM-MLLT10 |  |  |
| 51 | 11 | F | Acute Leukemia | Acute Leukemia of Ambigugous Lineage | NA | MLL/MLLT4 fusion: t(6;11)(q27;q23) | NA | KMT2A-AFDN | FISH | MCP-841 | Post Induction FCM-MRD 78% | Died | 1 | Acute Leukemia of Ambiguous lineage with KMT2A-AFDN |  |  |

**Supplementary Table 6: Table highlights genomic, immunophenotypic features and other validation details of fusions discovered by the lymphoid module of NARASIMHA. RT-PCR: reverse transcription-based PCR, MRD: measurable residual disease, ALL: acute lymphoblastic leukemia, CG: Conventional Karyotyping, NA: Not available**


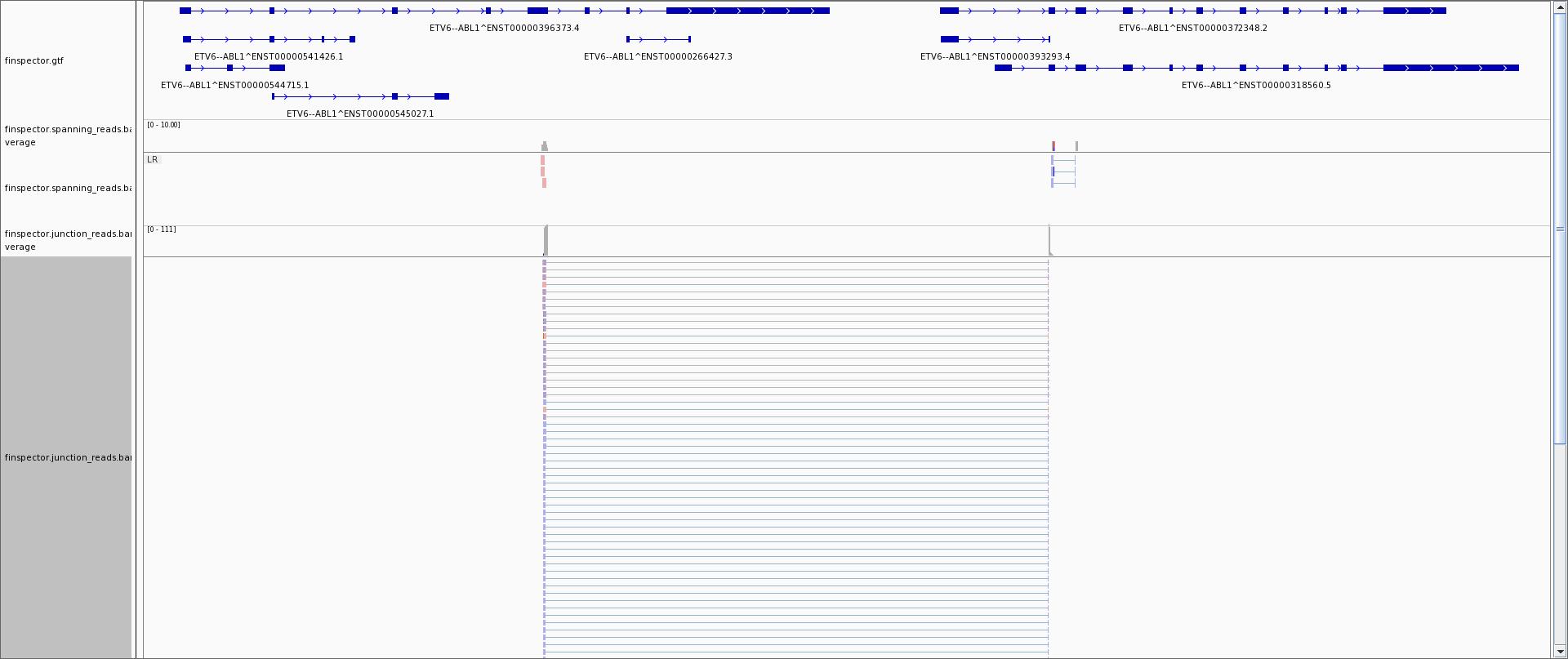
**Supplementary Figure 3: IGV image of an *ETV6-ABL1* CGF in a *BCR-ABL1* negative myeloproliferative neoplasm (Detailed case can be seen in supplementary table 5, Sr No 3)**

**
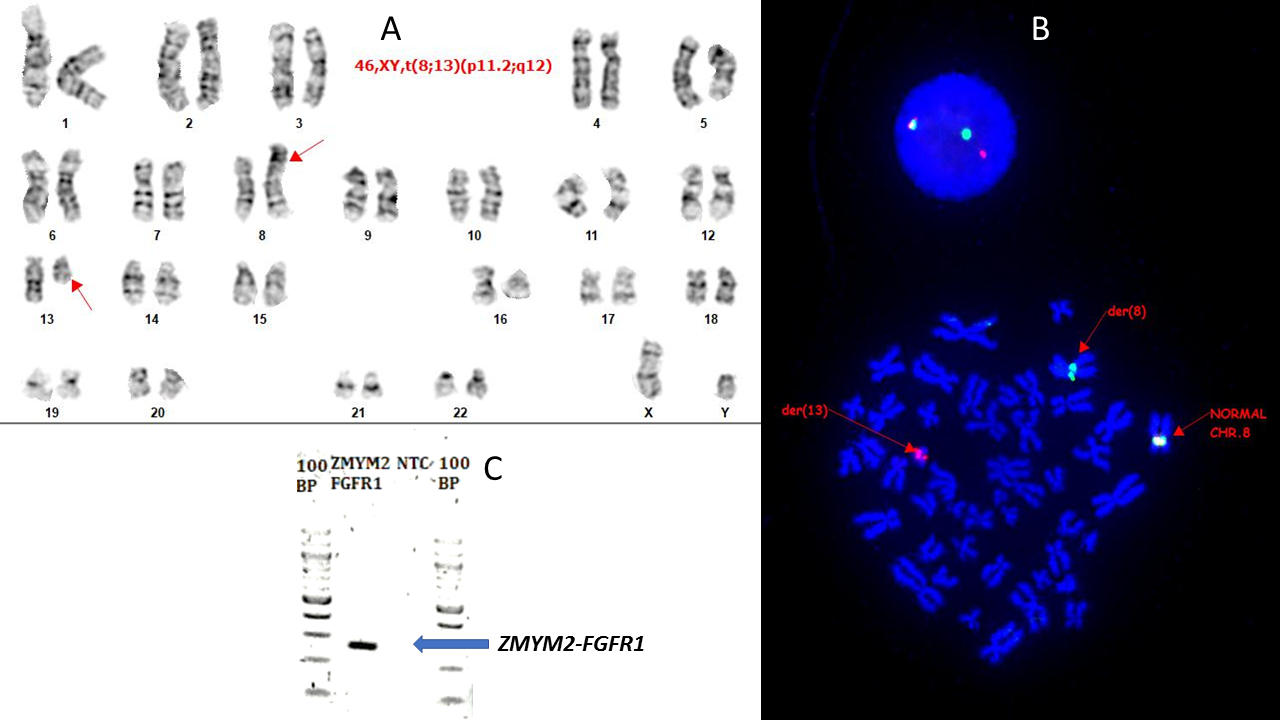
**

**Supplementary Figure 4: Composite image: Illustrative case of8p11 Myeloproliferative Syndrome detected by the lymphoid module of NARASIMHA and validated using A: Conventional Karyotyping, B: FISH, C:Reverse transcription based PCR (Detailed case can be seen in supplementary table 6, Sr No 10)**

**Supplementary References:**

1. Patkar N, et al. Development of a cost-effective 'duplexed' real-time PCR assay for minimal residual disease monitoring of chronic myeloid leukemia using locked nucleic acid probes. Int J Lab Hematol. 2016;38(6):e102-e6.

2. Shugay M, Ortiz de Mendibil I, Vizmanos JL, Novo FJ. Oncofuse: a computational framework for the prediction of the oncogenic potential of gene fusions. Bioinformatics. 2013;29(20):2539-46.

3. Tembhare P, et al. A novel and easy FxCycle violet based flow cytometric method for simultaneous assessment of DNA ploidy and six-color immunophenotyping. Cytometry A. 2016;89(3):281-91.

4. Narula G, et al. Clinicoepidemiological profiles, clinical practices, and the impact of holistic care interventions on outcomes of pediatric hematolymphoid malignancies - A 7-year audit of the pediatric hematolymphoid disease management group at Tata Memorial Hospital. Indian J Cancer. 2017;54(4):609-15.

5. Ghodke K, et al. CD19 negative precursor B acute lymphoblastic leukemia (B-ALL)-Immunophenotypic challenges in diagnosis and monitoring: A study of three cases. Cytometry B Clin Cytom. 2017;92(4):315-8.

6. Patkar N, et al. An integrated genomic profile that includes copy number alterations is highly predictive of minimal residual disease status in childhood precursor B-lineage acute lymphoblastic leukemia. Indian J Pathol Microbiol. 2017;60(2):209-13.

7. Patkar N, et al. Clinical impact of measurable residual disease monitoring by ultradeep next generation sequencing in NPM1 mutated acute myeloid leukemia. Oncotarget. 2018;9(93):36613-24.

8. Tembhare PR, et al. Evaluation of new markers for minimal residual disease monitoring in B-cell precursor acute lymphoblastic leukemia: CD73 and CD86 are the most relevant new markers to increase the efficacy of MRD 2016; 00B: 000-000. Cytometry B Clin Cytom. 2018;94(1):100-11.

9. Khattry N, et al. Long term clinical outcomes of adult hematolymphoid malignancies treated at Tata Memorial Hospital: An institutional audit. Indian J Cancer. 2018;55(1):9-15.

10. Patkar N, et al. Utility of Immunophenotypic Measurable Residual Disease in Adult Acute Myeloid Leukemia—Real-World Context. Frontiers in Oncology. 2019;9(450).

11. Tembhare PR, et al. A High-Sensitivity 10-Color Flow Cytometric Minimal Residual Disease Assay in B-Lymphoblastic Leukemia/Lymphoma Can Easily Achieve the Sensitivity of 2-in-10(6) and Is Superior to Standard Minimal Residual Disease Assay: A Study of 622 Patients. Cytometry B Clin Cytom. 2019.

12. Patkar N, et al. A novel machine-learning-derived genetic score correlates with measurable residual disease and is highly predictive of outcome in acute myeloid leukemia with mutated NPM1. Blood Cancer J. 2019;9(10):79.

13. Frederick L, Beardell F, Viswanatha DS. Novel BCR-ABL1 fusion identified by targeted next-generation sequencing in a patient with an atypical myeloproliferative neoplasm. Hum Pathol. 2014;45(8):1784-9.

14. Perna F, Abdel-Wahab O, Levine RL, Jhanwar SC, Imada K, Nimer SD. ETV6-ABL1-positive "chronic myeloid leukemia": clinical and molecular response to tyrosine kinase inhibition. Haematologica. 2011;96(2):342-3.

15. Arber DA, et al. The 2016 revision to the World Health Organization classification of myeloid neoplasms and acute leukemia. Blood. 2016;127(20):2391-405.

16. Lim JH, Jang S, Park CJ, Cho YU, Lee JH, Lee KH, et al. RANBP2-ALK fusion combined with monosomy 7 in acute myelomonocytic leukemia. Cancer Genet. 2014;207(1-2):40-5.

17. Jackson CC, Medeiros LJ, Miranda RN. 8p11 myeloproliferative syndrome: a review. Hum Pathol. 2010;41(4):461-76.

18. Gruber TA, Downing JR. The biology of pediatric acute megakaryoblastic leukemia. Blood. 2015;126(8):943-9.

19. Papaemmanuil E, et al. Genomic Classification and Prognosis in Acute Myeloid Leukemia. N Engl J Med. 2016;374(23):2209-21.

20. Xie W, Hu S, Xu J, Chen Z, Medeiros LJ, Tang G. Acute myeloid leukemia with t(8;16)(p11.2;p13.3)/KAT6A-CREBBP in adults. Ann Hematol. 2019;98(5):1149-57.

21. Meyer C, Burmeister T, Groger D, Tsaur G, Fechina L, Renneville A, et al. The MLL recombinome of acute leukemias in 2017. Leukemia. 2018;32(2):273-84.

22. Sanders MA, et al. RNA sequencing reveals a unique fusion of the lysine (K)-specific methyltransferase 2A and smooth muscle myosin heavy chain 11 in myelodysplastic syndrome and acute myeloid leukemia. Haematologica. 2015;100(1):e1-3.

23. Taketani T, Taki T, Ono R, Kobayashi Y, Ida K, Hayashi Y. The chromosome translocation t(7;11)(p15;p15) in acute myeloid leukemia results in fusion of the NUP98 gene with a HOXA cluster gene, HOXA13, but not HOXA9. Genes Chromosomes Cancer. 2002;34(4):437-43.

24. Shand JC. One giant leap for pediatric AMKL. Blood. 2016;127(26):3299-300.

25. Niktoreh N, et al. Mutated WT1, FLT3-ITD, and NUP98-NSD1 Fusion in Various Combinations Define a Poor Prognostic Group in Pediatric Acute Myeloid Leukemia. J Oncol. 2019;2019:1609128.

26. Potenza L, et al. A t(11;20)(p15;q11) may identify a subset of nontherapy-related acute myelocytic leukemia. Cancer Genet Cytogenet. 2004;149(2):164-8.

27. Savage NM, Kota V, Manaloor EJ, Kulharya AS, Pierini V, Mecucci C, et al. Acute leukemia with PICALM-MLLT10 fusion gene: diagnostic and treatment struggle. Cancer Genet Cytogenet. 2010;202(2):129-32.

28. Mullighan CG. The molecular genetic makeup of acute lymphoblastic leukemia. Hematology Am Soc Hematol Educ Program. 2012;2012:389-96.

29. De Braekeleer E, Douet-Guilbert N, Rowe D, Bown N, Morel F, Berthou C, et al. ABL1 fusion genes in hematological malignancies: a review. Eur J Haematol. 2011;86(5):361-71.

30. Lilljebjorn H, Fioretos T. New oncogenic subtypes in pediatric B-cell precursor acute lymphoblastic leukemia. Blood. 2017;130(12):1395-401.

31. Gu Z, Churchman M, Roberts K, Li Y, Liu Y, Harvey RC, et al. Genomic analyses identify recurrent MEF2D fusions in acute lymphoblastic leukaemia. Nat Commun. 2016;7:13331.

32. Ohki K, et al. Clinical and molecular characteristics of MEF2D fusion-positive B-cell precursor acute lymphoblastic leukemia in childhood, including a novel translocation resulting in MEF2D-HNRNPH1 gene fusion. Haematologica. 2019;104(1):128-37.

33. Denk D, et al. PAX5-AUTS2: a recurrent fusion gene in childhood B-cell precursor acute lymphoblastic leukemia. Leuk Res. 2012;36(8):e178-81.

34. Gu Z, Churchman ML, Roberts KG, Moore I, Zhou X, Nakitandwe J, et al. PAX5-driven subtypes of B-progenitor acute lymphoblastic leukemia. Nat Genet. 2019;51(2):296-307.

35. Coyaud E, et al. Wide diversity of PAX5 alterations in B-ALL: a Groupe Francophone de Cytogenetique Hematologique study. Blood. 2010;115(15):3089-97.

36. Hirabayashi S, et al. ZNF384-related fusion genes define a subgroup of childhood B-cell precursor acute lymphoblastic leukemia with a characteristic immunotype. Haematologica. 2017;102(1):118-29.

37. Moorman AV. New and emerging prognostic and predictive genetic biomarkers in B-cell precursor acute lymphoblastic leukemia. Haematologica. 2016;101(4):407-16.

38. Zaliova M, et al. Characterization of leukemias with ETV6-ABL1 fusion. Haematologica. 2016;101(9):1082-93.

39. Reshmi SC, et al. Targetable kinase gene fusions in high-risk B-ALL: a study from the Children's Oncology Group. Blood. 2017;129(25):3352-61.

40. Schwab C, Harrison CJ. Advances in B-cell Precursor Acute Lymphoblastic Leukemia Genomics. Hemasphere. 2018;2(4):e53.

41. Liu Y, et al. The genomic landscape of pediatric and young adult T-lineage acute lymphoblastic leukemia. Nat Genet. 2017;49(8):1211-8.

42. Iacobucci I, et al. Truncating Erythropoietin Receptor Rearrangements in Acute Lymphoblastic Leukemia. Cancer Cell. 2016;29(2):186-200.

43. Tasian SK, Loh ML, Hunger SP. Philadelphia chromosome-like acute lymphoblastic leukemia. Blood. 2017;130(19):2064-72.

44. Patnaik MM, Ketterling RP, Tefferi A. FGFR1 rearranged hematological neoplasms - molecularly defined and clinically heterogeneous. Leuk Lymphoma. 2018;59(7):1520-2.

45. Tirado CA, et al. Novel JAK2 rearrangement resulting from a t(9;22)(p24;q11.2) in B-acute lymphoblastic leukemia. Leuk Res. 2010;34(12):1674-6.

46. Welsh SJ, Churchman ML, Togni M, Mullighan CG, Hagman J. Deregulation of kinase signaling and lymphoid development in EBF1-PDGFRB ALL leukemogenesis. Leukemia. 2018;32(1):38-48.
